# Acquisition of suppressive function by conventional T cells limits anti-tumor immunity upon T_reg_ depletion

**DOI:** 10.1126/sciimmunol.abo5558

**Published:** 2023-12-15

**Authors:** Sarah K. Whiteside, Francis M. Grant, Giorgia Alvisi, James Clarke, Leqi Tang, Charlotte J. Imianowski, Baojie Zhang, Alexander C. Evans, Alexander J. Wesolowski, Alberto G. Conti, Jie Yang, Sarah N. Lauder, Mathew Clement, Ian R. Humphreys, James Dooley, Oliver Burton, Adrian Liston, Marco Alloisio, Emanuele Voulaz, Jean Langhorne, Klaus Okkenhaug, Enrico Lugli, Rahul Roychoudhuri

**Affiliations:** 1 Department of Pathology, University of Cambridge, Tennis Court Road, CB2 1QP, UK; 2 Immunology Programme, Babraham Institute, Babraham Research Campus, Cambridge, Cambridgeshire, CB22 3AT, UK; 3 Laboratory of Translational Immunology, IRCCS Humanitas Research Hospital, Via Manzoni 56, 20089 Rozzano – Milan, Italy; 4 La Jolla Institute for Allergy and Immunology, La Jolla, CA; 5 Division of Infection and Immunity/System Immunity University Research Institute, Cardiff University, Cardiff CF14 4XN, UK; 6 Department of Biomedical Sciences, Humanitas University, Via Rita Levi Montalcini 4, 20072 Pieve Emanuele – Milan, Italy; 7 Division of Thoracic Surgery, IRCCS Humanitas Research Hospital, Via Manzoni 56, 20089 Rozzano – Milan, Italy; 8 The Francis Crick Institute, 1 Midland Road, London NW1 1AT

## Abstract

Regulatory T (T_reg_) cells contribute to immune homeostasis but suppress immune responses to cancer. Strategies to disrupt T_reg_ cell-mediated cancer immunosuppression have been met with limited clinical success, but the underlying mechanisms for this failure are poorly understood. By modeling T_reg_ cell-targeted immunotherapy in mice, we find that CD4^+^ Foxp3^−^ conventional T (T_conv_) cells acquire suppressive function upon depletion of Foxp3^+^ T_reg_ cells, limiting therapeutic efficacy. Foxp3^−^ T_conv_ cells within tumors adopt a T_reg_ cell-like transcriptional profile upon ablation of T_reg_ cells and have a similar capacity to suppress T cell activation and proliferation *ex vivo*. Suppressive activity is enriched among CD4^+^ T_conv_ cells marked by expression of C-C motif receptor 8 (CCR8), which are found in mouse and human tumors. Upon T_reg_ cell depletion, CCR8^+^ T_conv_ cells undergo systemic and intratumoral activation and expansion, and mediate IL-10 dependent suppression of anti-tumor immunity. Consequently, conditional deletion of *Il10* within T cells augments anti-tumor immunity upon T_reg_ cell-depletion in mice, and antibody blockade of IL-10 signaling synergizes with T_reg_ cell depletion to overcome treatment resistance. These findings reveal a secondary layer of immunosuppression by T_conv_ cells released upon therapeutic T_reg_ cell depletion and suggest that broader consideration of suppressive function within the T cell lineage is required for development of effective T_reg_ cell-targeted therapies.

## Introduction

Immune checkpoint blockade therapies targeting the inhibitory receptors PD-1 and CTLA-4 on T_conv_ cells have revolutionized the treatment of advanced cancer (1–5). However, only a minority of patients with a subset of cancers respond to existing therapies (6–8), necessitating development of mechanistically distinct modes of immunotherapy. T_reg_ cells play a critical role in suppressing endogenous and immunotherapy-driven anti-tumor immunity (9–14). High relative ratios of T_reg_ cells to CD4^+^ or CD8^+^ T_conv_ cells within tumors are associated with poor prognoses in patients with a variety of cancers, including ovarian cancer (15, 16), breast cancer (17), non-small cell lung carcinoma (18), hepatocellular carcinoma (19), renal cell carcinoma (20), pancreatic cancer (21), gastric cancer (22), cervical cancer (23), intrahepatic cholangiocarcinoma (24) and colorectal carcinoma (25). Foxp3^+^ T_reg_ cells also powerfully contribute to immunotherapy resistance, including to immune checkpoint inhibitor therapy (18, 26–28). There is intense medical interest in therapeutically depleting T_reg_ cells or modulating their immunosuppressive function in cancer patients.

Despite abundant experimental evidence of the immunosuppressive role of T_reg_ cells in cancer, T_reg_ cell-targeted therapies have had limited success in the clinic. Agents developed for depletion of T_reg_ cells in humans have included daclizumab (Zenapax), a monoclonal antibody against CD25 which is expressed highly on the surface of most T_reg_ cells, denikeukin difitox (Ontak), an IL-2:diphtheria toxin fusion protein that targets T_reg_ cells through their ability to bind IL-2, and mogamulizumab, a depleting monoclonal antibody against CCR4, which is expressed by high frequencies of tumor-infiltrating T_reg_ cells (29). Daclizumab therapy failed to enhance the efficacy of a dendritic cell vaccine in metastatic melanoma patients (30), and only modestly increased immune response parameters in patients with glioblastoma (31) and breast cancer (32), while denikeukin difitox treatment failed to induce clinical responses in metastatic melanoma patients (33). Mogamulizumab therapy lacked anti-tumor efficacy in advanced cancer patients (34), likely attributable to concomitant depletion of activated CD4^+^ and CD8^+^ T_conv_ cells expressing CCR4 (35). Lack of clinical efficacy despite depletion of T_reg_ cells in many cases indicates a need to discern the basis for treatment failure of T_reg_ cell-targeted therapies.

In this study, we sought to better understand mechanisms of treatment failure of T_reg_ cell-targeting cancer immunotherapies. We systematically evaluated the consequence of experimental T_reg_ cell ablation on T_conv_ cells within tumors. Whereas CD4^+^ and CD8^+^ T_conv_ cells were markedly transcriptionally distinct from T_reg_ cells under steady-state conditions, T_reg_ cell ablation caused T_conv_ cells to adopt a T_reg_ cell-like transcriptional profile, upregulating expression of molecules associated with T_reg_ cell suppressive function. Consistent with acquisition of a T_reg_ cell-like transcriptional profile, Foxp3^−^ T_conv_ cells from T_reg_ cell-depleted animals acquired a potent ability to suppress T_conv_ activation and proliferation *in vitro*, attributable to the expansion and activation of a subset of T_conv_ cells marked by expression of CCR8. This subset of suppressive T_conv_ cells was found to be enriched in both murine and human tumors, and its suppressive function was dependent upon IL-10. Consequently, conditional deletion of *Il10* specifically within T cells, and blockade of IL-10 receptor (IL-10R) signaling during T_reg_ cell depleting immunotherapy reversed treatment failure and resulted in enhanced tumor clearance. These findings indicate that compensatory suppression by T_conv_ cells limits efficacy of T_reg_ cell-targeted therapeutic depletion and suggests that broader consideration of suppressive activity within the T cell lineage will be required for development of more effective therapies.

## Results

### T_reg_ cell ablation causes T_conv_ cells to adopt a T_reg_ cell-like transcriptional profile

**Figure 1:**
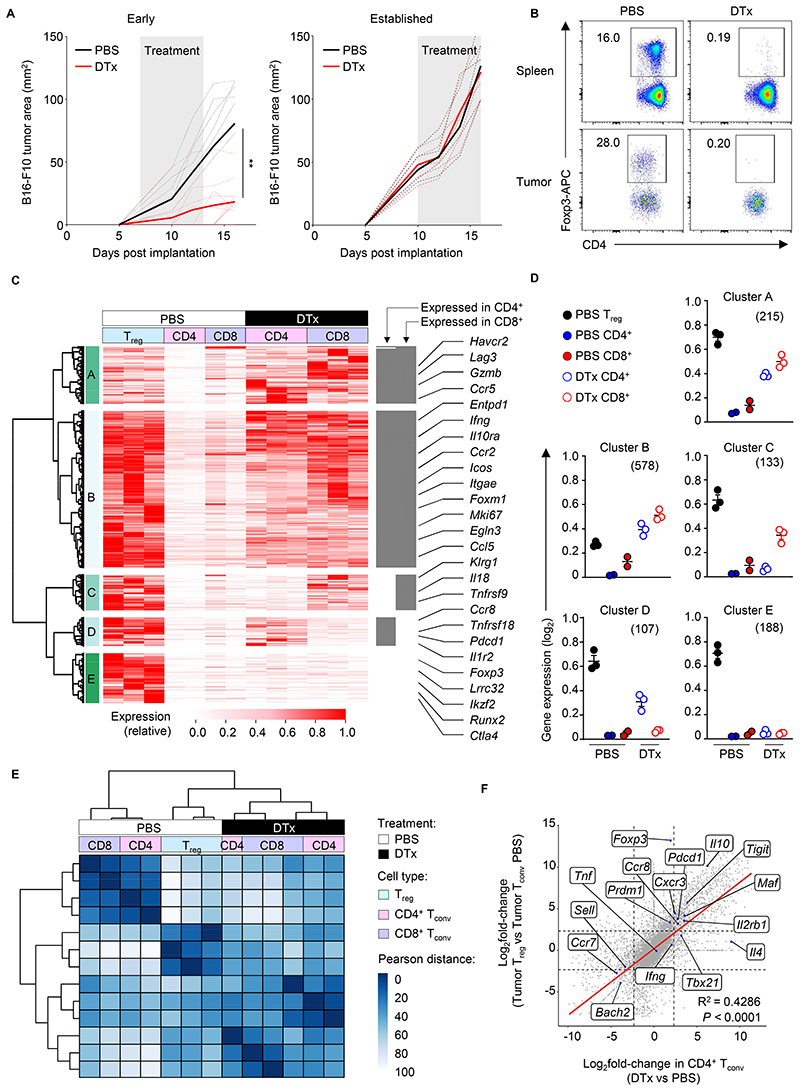
T_reg_ cell ablation causes T_conv_ cells to acquire transcriptional features of T_reg_ cells. (**A**) Tumor growth of B16-F10 tumors subcutaneously implanted into *Foxp3*^EGFP-DTR^ mice. Gray shading indicates time period over which PBS or DTx was administered (day 7-13 post-implantation, early disease; day 10-16 post-implantation, established). Dashed lines indicate individual mice. Solid line indicates average tumor area over time. Data representative of 4 individually repeated experiments, n > 5 **P < 0.01; two-tailed Mann–Whitney *U*-test. (**B**) Representative frequency of Foxp3^+^ T_reg_ cells among total CD4^+^ T cells within spleens (top) or tumors (bottom) of *Foxp3*^EGFP-DTR^ mice with established tumors administered PBS or DTx. (**C**) Heatmap showing the relative expression of transcripts upregulated in intratumoral T_reg_ cells compared with CD4^+^ T_conv_ cells (*q*<0.05; FC>4) in the indicated T cell subsets isolated at day 18 after implantation of B16-F10 tumors in *Foxp3*^EGFP-DTR^ animals administered PBS or DTx. Colors indicate expression normalized to row maxima. *x*-axis hierarchical clustering of intratumoral T_reg_ cell-expressed transcripts identifies 5 clusters of genes with distinct expression patterns. Gray bars to right of heatmap indicate expression greater than a third of the expression of given transcripts in intratumoral T_reg_ cells. (**D**) Average expression of genes within the 5 clusters identified in each T cell subset. **(E)** Heatmap showing pairwise Pearson distances between the global gene expression profiles of the indicated T cell subsets from B16-F10 tumor-bearing *Foxp3*^EGFP-DTR^ animals administered PBS or DTx. (**F**) Scatterplot comparing the global differences in gene expression between intratumoral T_reg_ cells and T_conv_ cells with transcriptional differences between CD4^+^ T_conv_ cells isolated from DTx versus PBS-treated animals. A highly significant correlation is observed indicating transcriptional convergence of intratumoral T_reg_ cells with T_conv_ cells in absence of T_reg_ cells. Data from 2-4 biological replicates isolated on independent days (C-F).

T_reg_ cell depletion has had limited success in cancer patients with advanced disease. To better understand mechanisms underlying treatment failure in the context of therapeutic T_reg_ cell ablation, we utilized *Foxp3*^EGFP-DTR^ mice, which express human diphtheria toxin receptor (DTR) and enhanced green fluorescent protein (EGFP) under the transcriptional control of the endogenous *Foxp3* gene. Administration of diphtheria toxin (DTx) to *Foxp3*^EGFP-DTR^ mice enables selective depletion of Foxp3^+^ T_reg_ cells (36). We subcutaneously implanted syngeneic B16-F10 melanoma cells into *Foxp3*^EGFP-DTR^ mice and ablated T_reg_ cells through administration of DTx. Early T_reg_ cell ablation (before tumors were palpable) resulted in incomplete rejection of primary tumors, whereas T_reg_ cell depletion in mice with established tumors had little discernible effect upon tumor growth (Fig. 1A), despite near complete ablation of Foxp3-expressing T_reg_ cells within the systemic and intratumoral compartments of DTx-treated mice (Fig. 1B).

To understand mechanisms of treatment resistance, we first examined the consequence of T_reg_ cell ablation on the transcriptional profiles of CD4^+^ and CD8^+^ T_conv_ cells within tumors. Surprisingly, although intratumoral *Foxp3*^EGFP−^ CD4^+^ and CD8^+^ T_conv_ cells were markedly transcriptionally distinct from *Foxp3*^EGFP+^ T_reg_ cells under steady-state conditions, T_reg_ cell depletion caused *Foxp3*^EGFP−^ T_conv_ cells to adopt a T_reg_ cell-like transcriptional profile. We noted that a large proportion of genes specifically enriched within tumor-associated T_reg_ cells compared with CD4^+^ T_conv_ cells (|FC| > 4, *q* < 0.05) under steady-state conditions were induced at high levels within CD4^+^ or CD8^+^ T_conv_ cells upon T_reg_ cell ablation (Fig. 1C and 1D; Data file S1). Clusters A and B comprised intratumoral T_reg_ cell-associated genes upregulated in both CD4^+^ and CD8^+^ T_conv_ cells upon T_reg_ cell ablation; Cluster C contained intratumoral T_reg_ cell-associated genes whose expression was upregulated exclusively in CD8^+^ T_conv_ cells; Cluster D included intratumoral T_reg_ cell-associated genes whose expression was upregulated exclusively in CD4^+^ T_conv_ cells; Cluster E comprised a limited set of T_reg_ cell-specific transcripts that were not expressed at high relative levels in CD4^+^ or CD8^+^ T_conv_ cells, including *Foxp3, Lrrc32, Ikzf2, Runx2*, and *Ctla4*. Similarly, a substantial fraction of transcripts highly expressed within intratumoral T_conv_ cells compared with T_reg_ cells were downregulated within T_conv_ cells upon T_reg_ cell ablation. (Fig. S1; Data file S2). Consistent with these observations, hierarchical clustering analysis of Pearson distances between global transcriptional profiles of samples revealed that intratumoral CD4^+^ and CD8^+^ T_conv_ cells from T_reg_ cell-depleted animals clustered more strongly with T_reg_ cells than T_conv_ cells from T_reg_ cell-sufficient animals (Fig. 1E). Moreover, differences in gene expression between CD4^+^ T_conv_ cells in the absence versus presence of T_reg_ cells were significantly positively correlated with differences in gene expression between intratumoral T_reg_ cells and CD4^+^ T_conv_ cells from T_reg_ cell-sufficient animals (Fig. 1F). Collectively, these results show that intratumoral T_conv_ cells adopt a T_reg_ cell-like transcriptional profile upon experimental ablation of T_reg_ cells *in vivo*.

### Ablation of T_reg_ cells promotes the induction of T_conv_ cell suppression

**Figure 2:**
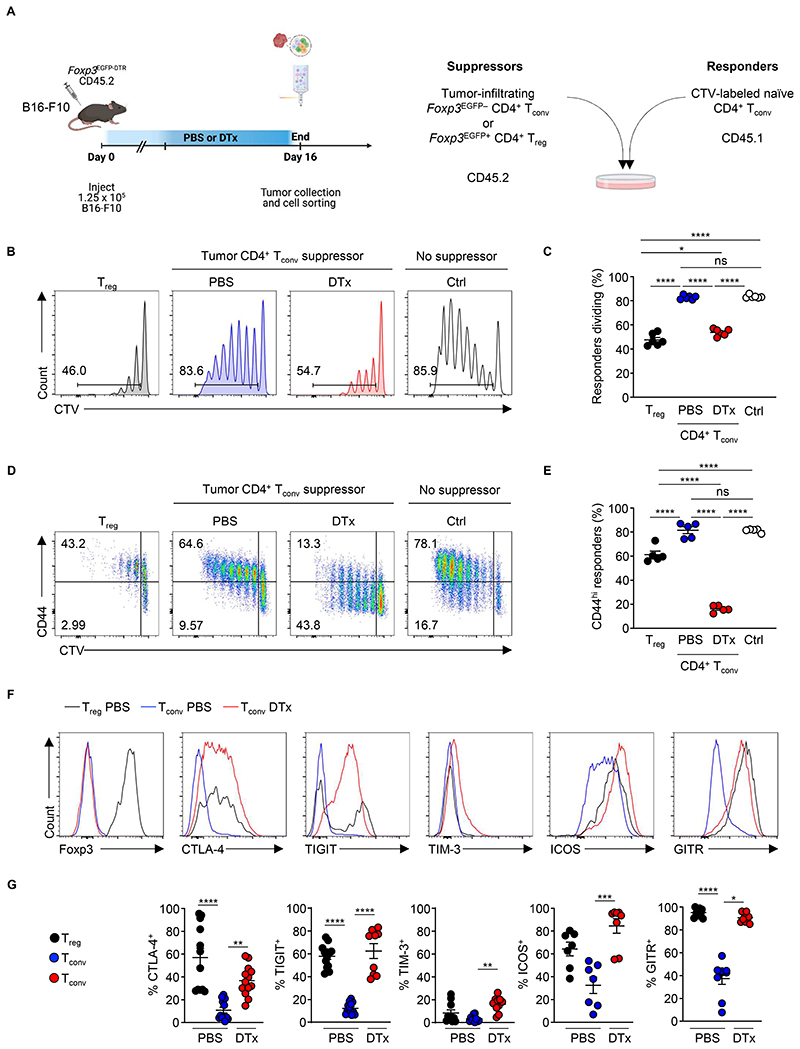
T_reg_ cell ablation promotes induction of suppressive function by CD4^+^ T_conv_ cells. (**A**) Experimental schema. B16-F10 cells were subcutaneously implanted into *Foxp3*^EGFP-DTR^ CD45.2^+^ mice and administered PBS or DTx on day 7, 9, 11, and 13. Cells were harvested from tumors of PBS- and DTx-treated animals at day 16 post-implantation and used in suppression assays. (**B** and **C**) *In vitro s*uppression assay. Proliferation of naïve CD45.1^+^ CD4^+^ T_conv_ responder (T_resp_) cells 4 days cultured at a 4:1 ratio with indicated suppressor cell populations (*Foxp3*^EGFP−^ T_conv_ cells from tumors of T_reg_-replete (PBS) or T_reg_-depleted (DTx) mice, or GFP^+^ T_reg_ cells from tumors of T_reg_-replete mice). Representative histograms and replicate measurements of proliferation dye dilution at day 4 post-stimulation gated on CD45.1^+^ T_resp_ cells shown. T_resp_ cell proliferation in the absence of a suppressor cell population was used as a control. Suppressor cells were co-cultured with responder T (Tresp) cells at a ratio of 1:4, with 2.5×10^4^ suppressor CD4^+^ T_reg_ or T_conv_ cells co-cultured with 1×10^5^ Tresp cells in the presence of 5.0×10^4^ antigen-presenting cells (APC). Data are representative of > 4 independently repeated experiments, n > 5 per group; ordinary one-way ANOVA, Tukey’s multiple comparisons. (**D** and **E**) Representative histograms and replicate measurements of CD44 expression by CD45.1^+^ T_resp_ cells incubated with indicated suppressor cell populations. (**F-G**) Representative flow cytometry and replicate measurements of the expression of the indicated proteins by intratumoral T_reg_ cells and CD4^+^ T_conv_ cells isolated at day 16 after implantation of B16-F10 tumors in *Foxp3*^EGFP-DTR^ mice treated with PBS or DTx. Data are representative of > 3 independently repeated experiments, n > 7 per group. ****P <0.0001; ns, not significant; one-way ANOVA Kruskal-Wallis, Dunn’s multiple comparisons test. Error bars show standard error of the mean (s.e.m.)

T_reg_ cells suppress the proliferation of naïve T_conv_ cells when co-cultured *in vitro* (37–39). Given their acquisition of a T_reg_ cell-like transcriptional profile, we asked whether T_conv_ cells develop suppressive function upon depletion of T_reg_ cells. To test this, we purified *Foxp3*^EGFP−^ CD4^+^ T_conv_ cells or *Foxp3*^EGFP+^ CD4^+^ T_reg_ cells by FACS from B16-F10-tumor bearing *Foxp3*^EGFP-DTR^ mice and incubated them with congenically distinct naïve CD4^+^ T_conv_ cells *in vitro* (Fig. 2A, Fig. S2). Strikingly, CD4^+^ T_conv_ cells from the tumors of mice whose T_reg_ cells had been ablated by administration of DTx profoundly suppressed the proliferation of naïve CD4^+^ T cell responders compared with CD4^+^ T_conv_ cells from tumors of animals with intact T_reg_ cell populations (Fig. 2B and C). The level of suppression was only marginally less than the level of suppressive activity of a similar number of intratumoral Foxp3^+^ T_reg_ cells. We observed lower levels of suppressive activity among splenic T_conv_ cells (Fig. S3A-B), suggesting that suppressive function was enriched in the tumor. In addition to suppressing T cell proliferation, T_conv_ cells from tumors of T_reg_ cell-depleted animals suppressed stimulation-driven induction of the activation marker CD44 on responder cells in contrast to T_conv_ cells from tumors of non-T_reg_ cell-depleted animals (Fig. 2D and E). T_conv_ cells from T_reg_ cell-depleted animals expressed similar levels of the co-inhibitory molecules TIGIT, TIM-3 and GITR to intratumoral T_reg_ cells (Fig. 2F and G). They also expressed higher levels of CTLA-4 and ICOS compared to CD4^+^ T_conv_ cells from tumors with intact T_reg_ cell populations. Acquisition of suppressive function by T_conv_ cells was not an artefact of DTx treatment, since administration of DTx to *Foxp3*^EGFP-DTR^ and control *Foxp3*^EGFP^ mice resulted in induction of potent suppressive activity only among CD4^+^ T_conv_ cells from *Foxp3*^EGFP-DTR^ animals, whose T_reg_ cells are sensitive to DTx treatment (Fig. S3C-D). Thus, upon T_reg_ cell ablation, CD4^+^ T_conv_ cells within tumors acquire transcriptional and functional characteristics of T_reg_ cells.

### T_reg_ cell depletion results in activation and expansion of CCR8^+^ T_conv_ cells

**Figure 3:**
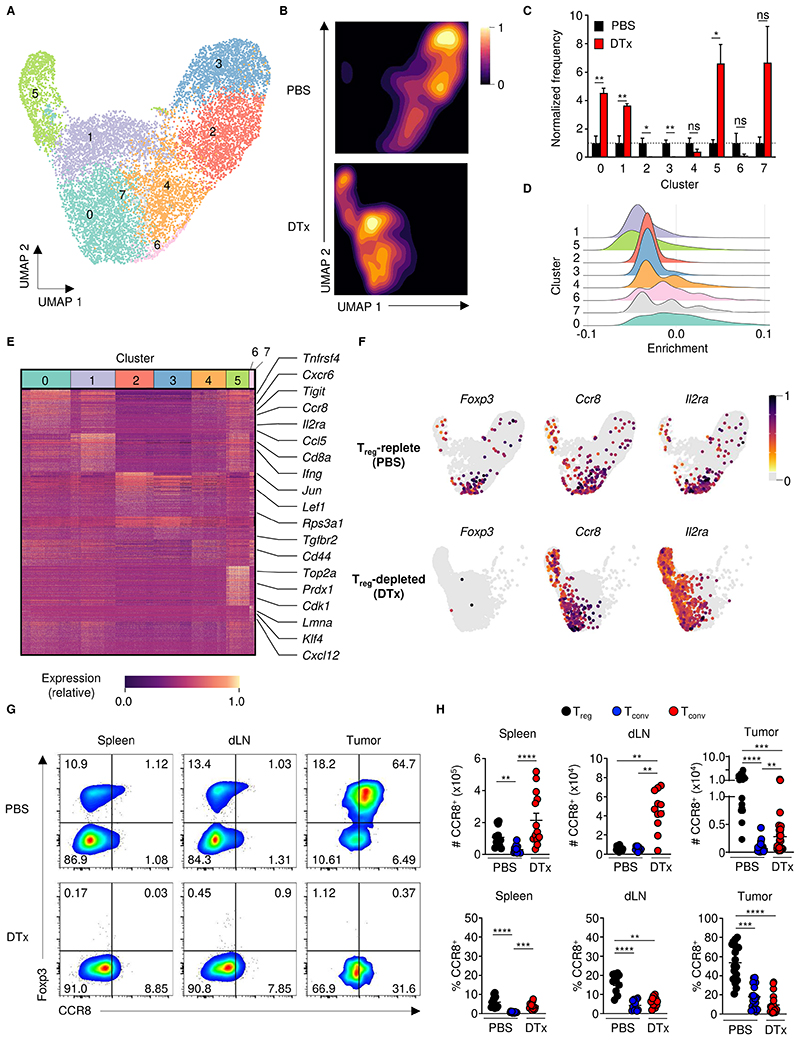
T_reg_ cell ablation promotes the expansion of tumor-infiltrating CCR8^+^ T_conv_ cells. (**A**) Uniform manifold approximation and projection (UMAP) of scRNA-Seq of TCRβ^+^ cells isolated at day 16 after implantation of B16-F10 tumors in *Foxp3*^EGFP-DTR^ mice treated with PBS or DTx on days 7, 9, 11 and 13. (**B**) Density plots showing change in distribution of cells within tumors of T_reg_-replete (PBS) and T_reg_-depleted (DTx) animals. (**C**) Relative frequency of cells within each cluster normalized to their average ratio among PBS animals. N = 3 biological replicates per group. (**D**) Average enrichment of expression of the genes in Cluster D from Fig. 1C across scRNA-Seq clusters, (n=3, unpaired two-tailed Student’s *t*-test, *P < 0.05, **P < 0.01). (**E**) Heatmap showing the expression of differentially upregulated genes in each cluster identified. (**F**) UMAP plots showing expression of indicated genes within T cells of tumors from tumor-bearing PBS- or DTx-treated *Foxp3*^EGFP-DTR^ animals. (**G**) Representative flow cytometry and (**H**) replicate measurements of total counts (top) and the frequency (bottom) of CCR8^+^ of CD4^+^ T_conv_ cells from spleens, draining lymph nodes (dLN) and tumors of B16-F10 tumor-bearing *Foxp3*^EGFP-DTR^ mice treated with PBS or DTx. Data are representative of 3 independently repeated experiments (**G** and **H**). Numbers in gates show percentages. n > 10. one-way ANOVA Kruskal-Wallis, Dunn’s multiple comparisons test. **P <0.01, ***P < 0.001, ****P < 0.0001; Error bars show standard error of the mean (s.e.m.)

To understand whether changes in the transcriptional and functional properties of bulk populations of T_conv_ cells in the absence of T_reg_ cells were driven by specific subpopulations, we performed scRNA-Seq of bulk T cell populations sorted by FACS from B16-F10 melanoma tumors of DTx- and PBS-treated *Foxp3*^EGFP-DTR^ animals at day 16 after tumor implantation. Single-cell gene expression data were clustered using Seurat and global transcriptional differences between cells were visualized in two-dimensional space using Uniform Manifold Approximation and Projection (UMAP). *k*-means clustering revealed the presence of 8 transcriptionally distinct clusters of cells (Fig. 3A). Clusters 2 and 3 were enriched in control samples, whereas Clusters 0, 1, 5 and 7 were enriched among T cells from tumors of T_reg_ cell-depleted animals (Fig. 3B and C). Enrichment analysis was used to determine which cell cluster was most responsible for the induction of T_reg_ cell-like gene expression within bulk RNA-Seq profiles of T_conv_ cells from T_reg_ cell-depleted animals. This revealed that Cluster 0 (present at a ~3:1 ratio in the DTx treatment condition) was most enriched in genes specifically upregulated by CD4^+^ T_conv_ cells upon DTx treatment (Fig. 3D). To define surface markers which would enable isolation of cells of Cluster 0, we performed an analysis of uniquely upregulated transcripts within each cluster. This analysis revealed that Cluster 0 cells express transcripts associated with T cell activation but which are also highly expressed by T_reg_ cells, including *Il2ra, Tigit* and *Tnfrsf4* (Fig. 3E and Data file S3). Strikingly, *Ccr8* mRNA expression was also upregulated in Cluster 0 cells upon depletion of T_reg_ cells, which was notable since we and others have shown that chemokine (C-C motif) receptor 8 (CCR8) marks highly suppressive T_reg_ cells under steady-state conditions within both murine and human tumors (40–44). A focused analysis of the CD4^+^ T cells in Cluster 0 revealed a sub-population of cells (subcluster 5) enriched in expression of transcripts encoding proteins associated with Th2 differentiation, including *Ccr8, Gata3, Maf* and *Il10*, and suppressive/co-inhibitory function, including *Pdcd1, Tigit, Il10* and *Lag3* (Fig. S4, Data file S4). Accordingly, an analysis of the distribution of cells expressing *Ccr8, Il2ra, Tigit* and *Tnfrsf4* revealed that while in T_reg_ cell-replete animals, these markers are largely expressed by intratumoral of *Foxp3*^EGFP+^ T_reg_ cells, they became expressed by a subset of *Foxp3*^EGFP−^ CD4^+^ T_conv_ cells upon T_reg_ cell depletion (Fig. 3F and Fig. S5).

We therefore analyzed the expression of CCR8 on the surface of T_reg_ and T_conv_ cells in tumors and lymphatics of *Foxp3*^EGFP-DTR^ animals treated with PBS or DTx (Fig. 3G and H). We found that T_reg_ cell depletion increased the absolute number of CCR8^+^ T_conv_ cells within all tissues analyzed, including tumors, draining lymph nodes (dLN) and spleen, whereas the relative frequency of CCR8^+^ cells among total CD4^+^ *Foxp3*^EGFP−^ T_conv_ cells was increased within the spleens of T_reg_ cell-depleted animals, but not within dLN and tumors due to the absolute expansion of other CD4^+^ *Foxp3*^EGFP−^ T_conv_ cells subsets in these tissues upon T_reg_ cell depletion.

### CCR8 expression marks highly suppressive T_conv_ cells within tumors

**Figure 4:**
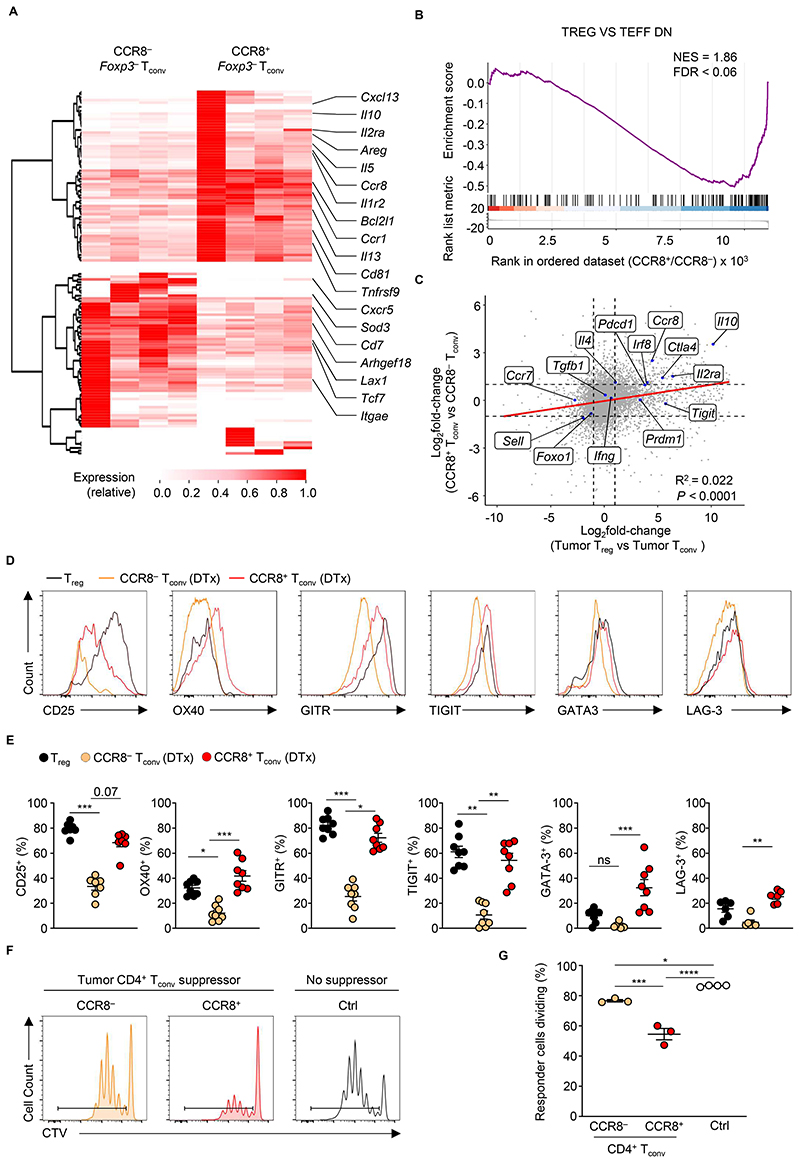
CCR8 marks highly suppressive T_conv_ cells. (**A**) Heatmap showing the relative expression of differentially expressed genes between intratumoral CCR8^+^ and CCR8^−^ CD4^+^ T_conv_ cells (*q*<0.05; |FC|>3) isolated at day 16 after subcutaneous implantation of B16-F10 tumors of T_reg_-depleted *Foxp3*^EGFP-DTR^ animals treated with DTx at days 7, 9, 11 and 13. Data from 4 biological replicates isolated on the same day. (**B**) Gene-set enrichment analysis (GSEA) demonstrating a negative enrichment of genes upregulated in *Foxp3*^−^ T_conv_ cells *vs* *Foxp3*^+^ T_reg_ cells among CCR8^+^ T_conv_ cells compared with CCR8^−^ T_conv_ cells isolated from tumors of DTx-treated *Foxp3*^EGFP-DTR^ mice. (**C**) Scatterplot comparing global changes in gene expression between intratumoral T_reg_ and T_conv_ cells with transcriptional differences between CCR8^+^ and CCR8^−^ CD4^+^ T_conv_ cells. (**D**) Representative flow cytometry and (**E**) replicate measurements of the expression of the indicated proteins from intratumoral T_reg_ cells and CCR8^+^ and CCR8^−^ CD4^+^ T_conv_ cells from tumors of B16-F10 tumor-bearing *Foxp3*^EGFP-DTR^ mice treated with PBS or DTx. Data are representative of 3 independently repeated experiments n > 4 one-way ANOVA Kruskal-Wallis, Dunn’s multiple comparisons test. *P < 0.05, **P <0.01, ***P < 0.001, ****P < 0.0001, ns, not significant. (**F**) Representative histograms and (**G**) replicate measurements of T_resp_ cells (naïve CD45.1^+^ CD4^+^ T_conv_ cells) incubated with intratumoral CD45.2^+^ TCRβ^+^ CD4^+^ GFP^−^ CCR8^−^ or CD45.2^+^ TCRβ^+^ CD4^+^ GFP^−^ CCR8^+^ suppressor T_conv_ cells isolated at day 16 after implantation of B16-F10 tumors in *Foxp3*^EGFP-DTR^ mice. Suppressor cells were incubated with responders at a ratio of 1:8, with 1.25×10^4^ suppressor CD4^+^ T_conv_ cells co-cultured with 1×10^5^ Tresp cells in the presence of 5×10^4^ APC. Cell proliferation of T_resp_ cells was analyzed after 4 days. Data are representative of 2 independently repeated experiments n > 3. ordinary one-way ANOVA, Tukey’s multiple comparisons. *P < 0.05; ***P < 0.001, ****P < 0.0001; Error bars show standard error of the mean (s.e.m.)

To better understand the identity of CCR8^+^ T_conv_ cells, we purified CCR8^+^ and CCR8^−^ CD4^+^ T_conv_ cells by FACS from B16-F10 tumors of DTX-treated *Foxp3*^EGFP-DTR^ mice and subjected them to bulk RNA-Seq. CCR8^+^ T_conv_ cells were enriched in transcripts encoding molecules associated with both T cell activation such as *Tnfrsf9* (encoding 4-1BB) and T_reg_ cell suppressive function, including *Il2ra, Areg*, and *Il10* (Fig. 4A and Data file S5). Interestingly, CCR8^+^ T_conv_ cells were not enriched in transcripts associated with suppressive type 1 regulatory T cells (Tr1) such as *Eomes, Gzmk, Itga2* (encoding CD49b), *Ccr5*, or *Cd226*, suggesting that they are distinct from Tr1 cells. Gene set enrichment analysis (GSEA) of global gene expression differences between CCR8^+^ and CCR8^−^ T_conv_ cells revealed a negative enrichment of genes upregulated in *Foxp3*^−^ T_conv_ cells *vs Foxp3^+^* T_reg_ cells among CCR8^+^ T_conv_ cells compared with CCR8^−^ T_conv_ cells (Fig. 4B). Consistently, we observed that global differences in gene expression between CCR8^+^ and CCR8^−^ T_conv_ cells were positively correlated with global differences in gene expression between intratumoral T_reg_ and T_conv_ cells (Fig. 4C), further suggesting that CCR8^+^ *Foxp3*^−^ T_conv_ cells possess a T_reg_ cell-like transcriptional profile.

We compared the phenotype of CCR8^−^ and CCR8^+^ CD4^+^ T_conv_ cells from tumors of mice whose T_reg_ cells had been depleted by DTx with that of T_reg_ cells. Like T_reg_ cells, we found that CCR8^+^ CD4^+^ T_conv_ cells expressed high levels of CD25, OX40, GITR, TIGIT and LAG-3 compared to CCR8^−^ CD4^+^ T_conv_ cells (Fig. 4D and E). CCR8^+^ T_conv_ cells also expressed increased levels of the transcription factor GATA3, suggesting that they possess a Th2-like differentiation state. To test whether the accumulation of CCR8^+^ cells within intratumoral T_conv_ cell populations following T_reg_ cell ablation accounts for their increased suppressive activity, we separately sorted CCR8^+^ and CCR8^−^ *Foxp3*^EGFP−^ T_conv_ cells from the tumors of DTx-treated *Foxp3*^EGFP-DTR^ mice and assessed their ability to suppress naïve T_conv_ cell proliferation *in vitro*. Notably, suppressive function was enriched within the CCR8^+^ T_conv_ cell fraction, which were more capable of restricting proliferation of responder cells compared to the CCR8^−^ T_conv_ cell fraction (Fig. 4F and G). Taken together, these results suggest that CCR8 expression marks a subset of highly activated and suppressive T_conv_ cells which accumulate systemically and within tumors upon T_reg_ cell depletion.

### CD4^+^ FOXP3^−^ CCR8^+^ T_conv_ cells are found within tumors of NSCLC patients

**Figure 5:**
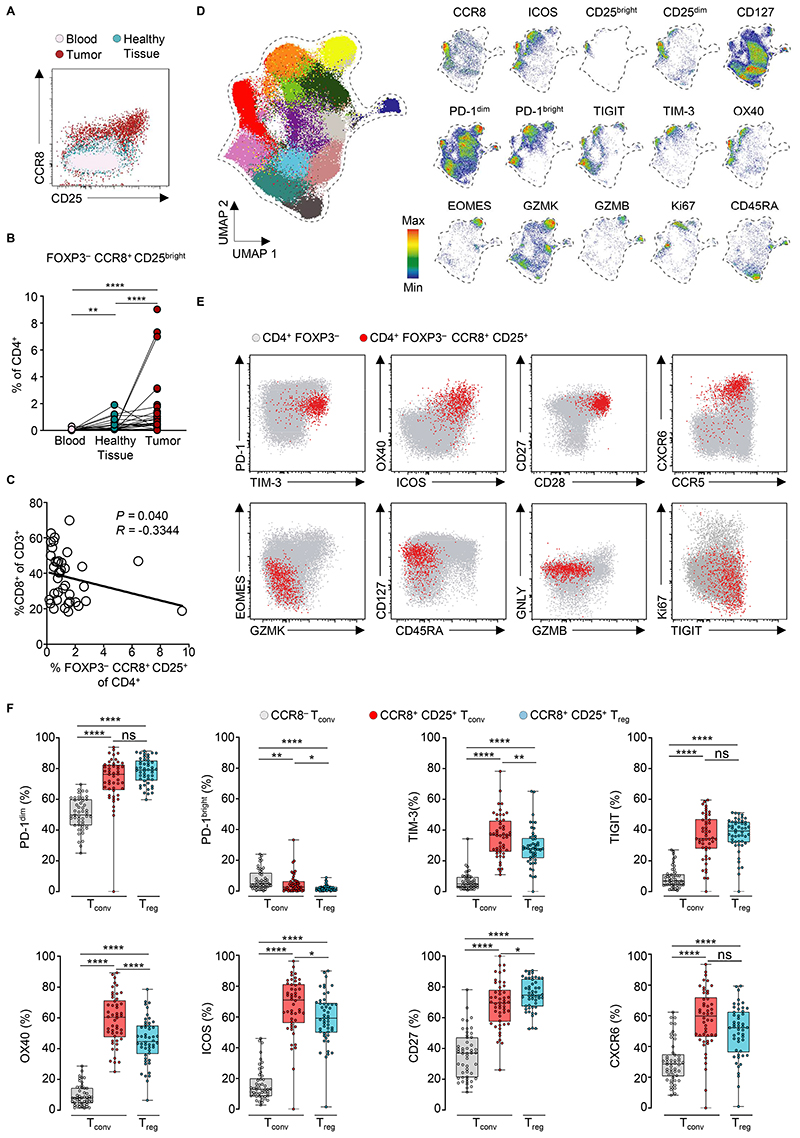
CCR8^+^ T_conv_ cells expressing high levels of CD25 are found within the tumors of NSCLC patients. (**A**) CCR8 and CD25 expression among FOXP3^−^ CD4^+^ T cells from representative samples from NSCLC patients (n=48). (**B**) Frequency of FOXP3^−^ CCR8^+^ CD25^bright^ cells among CD4^+^ T cells from the indicated patients’ samples. Lines indicate paired samples. (**C**) Correlation of the frequency of CD8^+^ T cells (of CD3^+^ T cells) with FOXP3^−^ CCR8^+^ CD25^bright^ cells (of CD4^+^ T cells) in tumors from patient samples. (**D**) UMAP analysis of concatenated CD4^+^ FOXP3^−^ T_conv_ cells. Colors depict cell clusters identified by Phenograph (k=500). Separate UMAP plots of relative marker expression by concatenated CD4^+^ T cells from tumors. (**E**) Representative frequency and (**F**) replicate measurements of indicated markers from patient samples, box plots show median and interquartile range (IQR). Bars indicate standard deviation. Dots depict values of a single tumor sample. **P < 0.01, ***P < 0.001, ****P < 0.0001; two-tailed Mann–Whitney *U*-test.

In order to determine if CCR8^+^ FOXP3^−^ T_conv_ cells are enriched in human tumors, we analyzed CD4^+^ T cells from 48 patents with non-small cell lung carcinoma (NSCLC) by flow cytometry (Fig. S6). Similar to our observations in mouse, CCR8^+^ FOXP3^−^ T_conv_ cells expressed high levels of CD25 and were significantly enriched in tumor tissue compared to healthy adjacent tissue and blood from the same patients (Fig. 5A and 5B). Moreover, the frequency of CCR8^+^ CD25^+^ FOXP3^−^ T_conv_ cells was inversely correlated with the frequency of cytotoxic CD8^+^ T cells within tumors (Fig. 5C). They also co-expressed the inhibitory receptors PD-1, TIGIT and TIM-3 (Fig. 5D and E). CD4^+^ FOXP3^−^ CCR8^+^ T cells displayed increased expression of the tissue-residency marker CXCR6 and co-stimulatory receptor CD27 compared to CCR8^−^ cells and their phenotype was largely overlapping with that of FOXP3^+^ T_reg_ cells (Fig. 5E and F). CD4^+^ FOXP3^−^ CCR8^+^ T cells lacked expression of EOMES and Granzyme K, providing further evidence that this cell type is distinct from Tr1 cells (Fig. 5E). The presence of CCR8^+^ FOXP3^−^ T_conv_ cells in human tumors under steady-state conditions and without T_reg_ cell depletion is consistent with our observations within murine tumors (Fig. 3H and I), which contain a population of CCR8^+^ CD4^+^ T_conv_ cells under basal conditions that undergo substantial expansion upon T_reg_ cell ablation.

### IL-10-dependent immune suppression by T_conv_ cells limits efficacy of T_reg_ cell depletion

**Figure 6:**
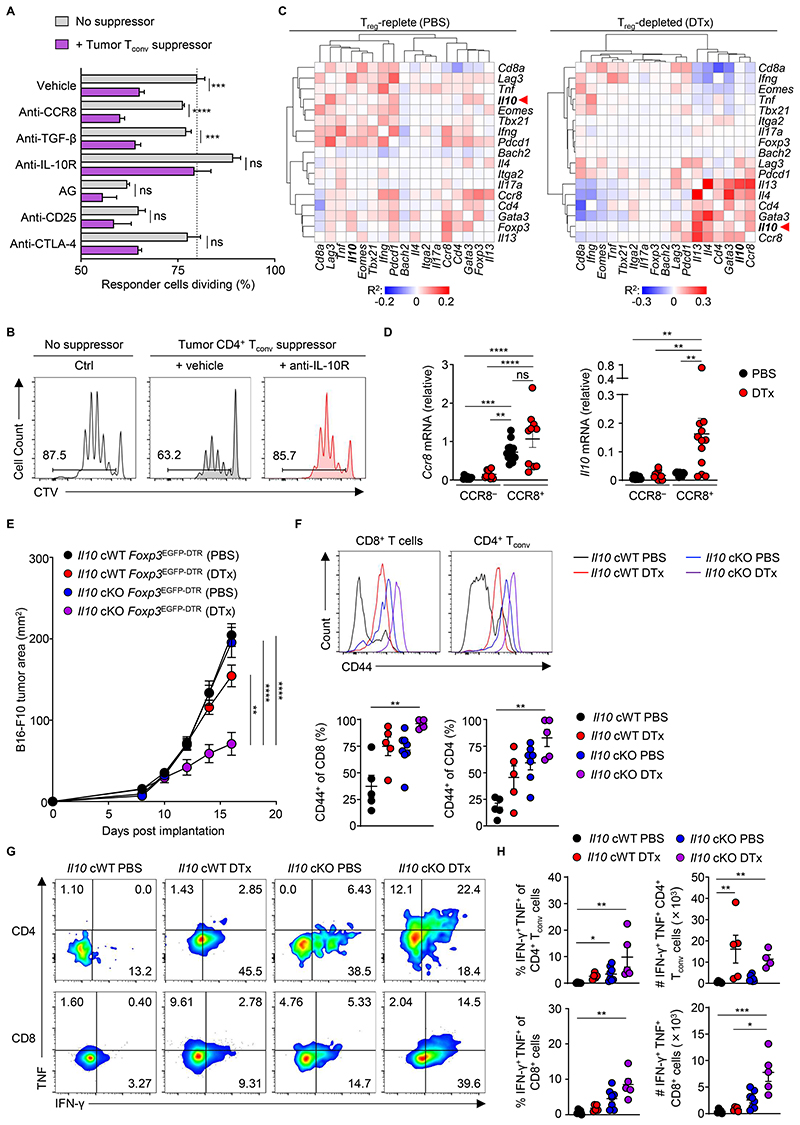
IL-10 production by CD4^+^ T_conv_ cells limits anti-tumor immunity upon T_reg_ cell depletion. (**A**) Screen to identify mechanisms of suppression by CD4^+^ T_conv_ cells from tumors of T_reg_-depleted animals. Proliferation of CTV-labeled naïve splenic CD4^+^ T_conv_ responder cells (T_resp_) cultured alone or at a ratio of 8:1 with CD4^+^ *Foxp3*^EGFP−^ T_conv_ suppressor cells (T_supp_) isolated at day 16 from B16-F10 tumors of DTx-treated *Foxp3*^EGFP-DTR^ animals. Cells were cultured alone (gray) or with suppressors (purple) and with indicated reagents. AG, aminoglutethimide. CD45.2^+^ GFP^−^ T_conv_ suppressor cells were co-cultured with 1.25×10^4^ suppressor CD4^+^ T_conv_ cells in the presence of 5.0×10^4^ antigen-presenting cells (APC). Data are representative of 2 independently repeated experiments n > 3. *P* values show significance of difference between no suppressor and suppressor (Student *t* test) and are Bonferroni corrected. (**B**) Representative frequency of dividing T_resp_ cells incubated with tumor CD4^+^ GFP^−^ T_conv_ cells in the presence of anti-IL-10R antibodies or vehicle. T_resp_ cells without tumor T_conv_ cells were used as a control. (**C**) Co-correlation between the expression of indicated genes within single cell gene expression profiles of T cells from tumors of PBS- or DTx-treated B16 tumor-bearing *Foxp3*^EGFP-DTR^ animals. Pearson correlation co-efficient values are indicated by color scale and genes are hierarchically clustered to identify clusters of co-expressed transcripts within T cell populations. scRNA-Seq data are representative of 3 biological replicates per group. (**D)** Measurement of *Ccr8* and *Il10* mRNA expression within CCR8^−^ and CCR8^+^ T_conv_ cells from PBS- and DTx-treated animals. Data representative of 3-4 biological replicates per group. ordinary one-way ANOVA, Tukey’s multiple comparisons. ns, not significant. (**E**) Tumor area of heterotopic B16-F10 melanoma tumors at indicated time-points following implantation into *Il10*^flox/flox^ *Cd4*^Cre^ *Foxp3*^EGFP-DTR^ or *Il10*^+/+^ *Cd4*^Cre^ *Foxp3*^EGFP-DTR^ control mice administered DTx or PBS from day 10-16 post-implantation. Data are representative of 2 independently repeated experiments, n > 7. ordinary one-way ANOVA, Tukey’s multiple comparisons. (**F**) Representative histograms (top) and replicate measurements (bottom) of the frequency of CD8^+^ CD44^+^ T cells and Foxp3^−^ CD4^+^ CD44^+^ T_conv_ cells from tumors of animals within indicated treatment groups. (**G**) Representative frequency and (**H**) replicate measurements of the frequency (top) and total counts (bottom) of CD8^+^ IFN-γ^+^ TNF^+^ T cells within tumors. Data are representative of 2 independently repeated experiments, n > 4, one-way ANOVA, Kruskal-Wallis, Dunn’s multiple comparisons test. *P < 0.05; **P < 0.01; ***P < 0.001, ****P < 0.0001. Error bars show standard error of the mean (s.e.m.)

We sought to understand how intratumoral CD4^+^ T_conv_ cells exert their suppressive function. Informed by the results of our transcriptional analyses, we screened for the involvement of candidate suppressive mechanisms by which T_conv_ cells from T_reg_ cell-depleted animals suppress T cell activation and proliferation *in vitro*. We tested whether blocking antibodies directed against CD25, CTLA-4, IL-10 receptor (IL-10R) and CCR8, neutralizing antibodies specific for transforming growth factor (TGF)-β, or pharmacological inhibition of steroid biosynthesis preferentially produced by Th2 cells using aminoglutethimide (AG) (45), are able to reverse the suppressive activity of intratumoral T_conv_ cells from T_reg_ cell-depleted animals. Importantly, while the proliferation of naïve CD4^+^ T cells was suppressed by Foxp3^−^ T_conv_ cells, these differences induced by the presence of suppressive Foxp3^−^ T_conv_ cells were abolished upon treatment of cells with anti-IL-10R (Fig. 6A and B).

Given these observations, we asked whether CCR8^+^ T_conv_ cells are primary producers of *Il10* mRNA upon depletion of T_reg_ cells. We first examined whether the expression of *Il10* mRNA is co-correlated with the expression of *Ccr8* mRNA, and therefore co-expressed within the same cells, using scRNA-Seq of T cells from tumors of T_reg_ cell-replete and - depleted animals. Since IL-10 is known to be produced by CD4^+^ T_R_1 cells which express LAG3 and CD49b (46–49), Eomes^+^ CD4^+^ T_conv_ cells (50–52), exhausted CD8^+^ T cells which express PD-1 (53) and CD4^+^ Th2 cells which express GATA3, IL-4 and IL-13 (54, 55), we included the genes encoding these and other markers in our co-correlation analysis. We found that under steady-state (T_reg_ cell-replete) conditions, *Il10* formed a predominant co-correlation cluster with *Cd8a, Pdcd1, Ifng, Tnf*, and *Eomes* but also a smaller cluster containing *Gata3* and *Foxp3*, suggesting that CD8^+^ T cells in differential states of exhaustion, and Th2-like T_reg_ cells are a predominant source of IL-10 (Fig. 6C). However, we found that T_reg_ cell depletion resulted in a striking change in the co-correlation relationship of *Il10* mRNA with the other genes examined, forming a predominant cluster of co-correlated genes containing *Cd4, Ccr8, Il13, Il14* and *Gata3*. These results suggested that upon T_reg_ cell ablation, the source of *Il10* shifts to the previously identified CD4^+^ CCR8^+^ T_conv_ cell subset with Th2-like characteristics, and that CD4^+^ CCR8^+^ T_conv_ cells undergo activation into *Il10-*expressing cells. To confirm this, we sorted CCR8^−^ and CCR8^+^ T_conv_ cells from tumors of T_reg_ cell-replete and -depleted animals and subjected them to qRT-PCR. We found that *Il10* mRNA expression was enriched among CCR8^+^ T_conv_ cells from T_reg_ cell-depleted animals compared with both CCR8^−^ cells from T_reg_ cell-depleted animals and CCR8^+^ or CCR8^−^ cells from T_reg_ cell-replete animals (Fig. 6D). These findings supported the hypothesis that CCR8^+^ T_conv_ cells become the predominant source of T cell-expressed IL-10 upon T_reg_ cell depletion.

We therefore asked whether T_reg_ cell depletion triggers induction of IL-10-dependent suppressive activity among Foxp3^−^ T_conv_ cells, limiting efficacy of T_reg_ cell depletion *in vivo*. We had observed that T_reg_ cell depletion was ineffective at significantly reducing growth of established B16 tumors, while T_reg_ cell depletion during early disease delayed tumor growth but was ineffective at inducing complete responses (Fig. 1A). To test whether IL-10 production by T_conv_ cells is responsible for resistance to T_reg_ cell-depleting therapy *in vivo*, we generated *Il10*^flox/flox^ *Cd4*^Cre^ *Foxp3*^EGFP-DTR^ and littermate *Cd4*^Cre^ *Foxp3*^EGFP-DTR^ (IL-10 proficient) control mice. This allowed us to examine the effect of T_reg_ cell depletion in animals whose remaining T cells can or cannot produce IL-10. Since IL-10 ablation has been shown to promote tumor growth under steady-state conditions (56, 57), we subcutaneously implanted with B16-F10 cells into *Il10*^flox/flox^ *Cd4*^Cre^ *Foxp3*^EGFP-DTR^ and littermate *Cd4*^Cre^ *Foxp3*^EGFP-DTR^ control animals and selected animals with tumors of similar size (range 12 - 64 mm^2^) at day 10 after tumor implantation for randomization to treatment groups (PBS or DTx). We found that conditional deletion of IL-10 within T cells resulted in loss of resistance to T_reg_ cell depletion, as indicated by reduced tumor growth when T_reg_ cells were ablated in animals lacking T cell-restricted IL-10 expression, but not when either condition was present alone (Fig. 6E). There was also an increase in expression of the activation marker CD44 on CD4^+^ T_conv_ cells and CD8^+^ T cells from animals bearing a conditional deletion of *Il10* and whose T_reg_ cells had been ablated (Fig. 6F). T_reg_ cell ablation in animals bearing a conditional deletion of *Il10* within T cells was associated with increased frequencies of CD8^+^ T cells and CD4^+^ T_conv_ cells expressing the cytokines IFN-γ and TNF (Fig. 6G and H). These results demonstrate a critical role for T cell-produced IL-10 in resistance to T_reg_ cell depletion.

### Blockade of IL-10 signaling synergizes with T_reg_ cell depletion to induce robust anti-tumor immune responses

**Figure 7:**
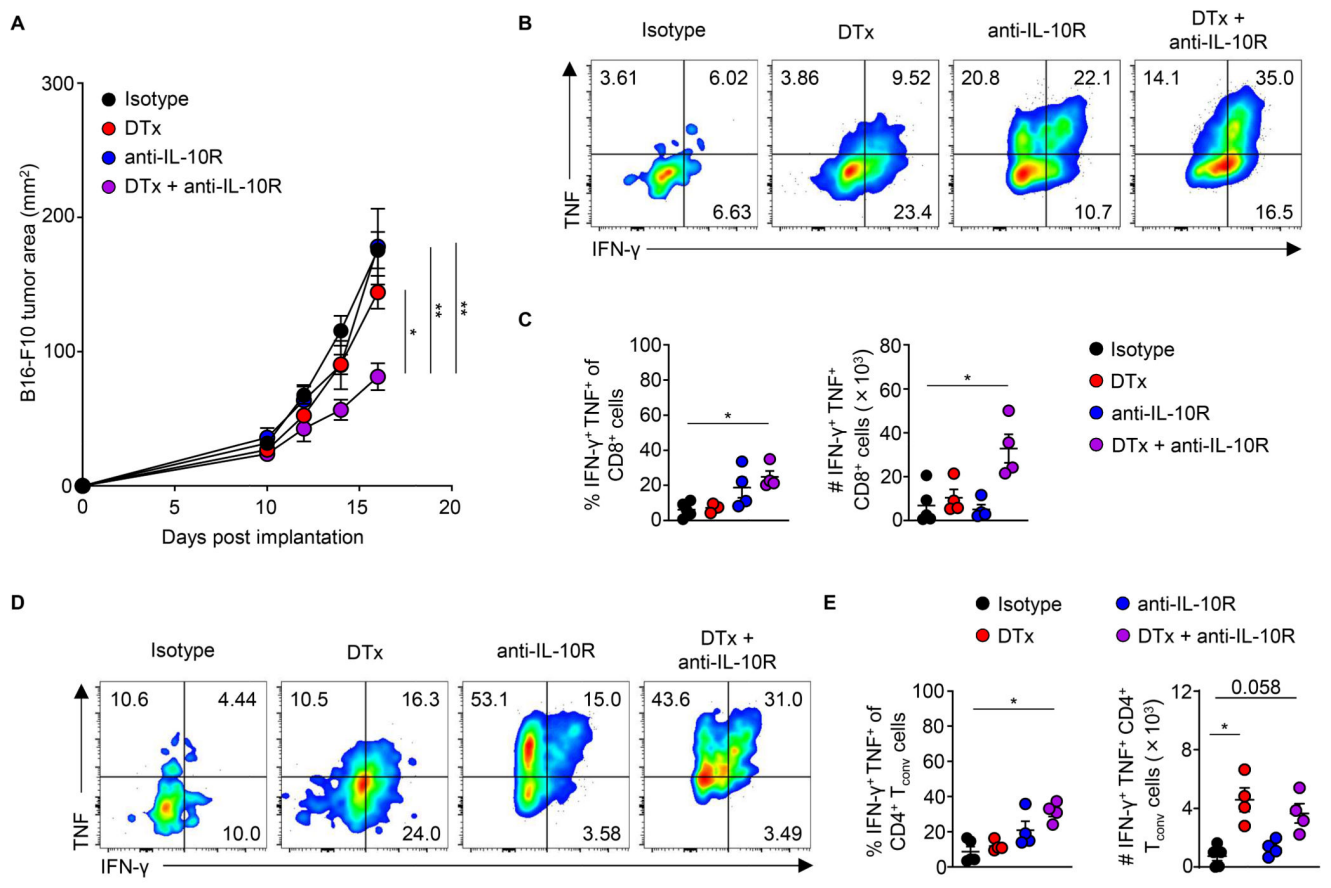
Blockade of IL-10 signaling synergizes with T_reg_ cell-depletion to drive potent anti-tumor immune responses. (**A**) Tumor area of B16-F10 melanoma tumors at indicated time-points following implantation into *Foxp3*^EGFP-DTR^ animals administered indicated combinations of DTx and anti-IL-10R or control reagents from days 10-16 after tumor implantation. Data are representative of 2 independently repeated experiments. n > 10, ordinary one-way ANOVA, Tukey’s multiple comparisons. *P < 0.05 **P < 0.01; (**B**) Representative frequency and (**C**) replicate measurements of the frequency and total counts of CD8^+^ IFN-γ^+^ TNF^+^ T cells from tumors. n > 3, one-way ANOVA, Kruskal-Wallis, Dunn’s multiple comparisons test., *P < 0.05. (**D**) Representative frequency and (**E**) replicate measurements of the frequency and total counts of Foxp3^−^ CD4^+^ IFN-γ^+^ TNF^+^ T cells from tumors. Data from > 2 independent biological replicates. n > 3, Kruskal-Wallis, Dunn’s multiple comparisons test., *P < 0.05; Error bars show standard error of the mean (s.e.m.)

We next asked whether antibody blockade of IL-10 signaling synergizes with T_reg_ cell depletion *in vivo*. We tested whether blockade of IL-10R using anti-IL-10R antibodies reverses resistance to T_reg_ cell-depleting therapy. We found that late T_reg_ cell depletion or IL-10R blockade alone failed to drive significant reduction in tumor growth, whereas their combination resulted in potent tumor regression (Fig. 7A). Moreover, IL-10 blockade synergized with early T_reg_ cell ablation to induce complete responses in a proportion of animals receiving combined therapy (Fig. S7). We found that IL-10R blockade both alone and in combination with T_reg_ cell depletion increased the ratio of CD8^+^ T cells expressing IFN-γ and TNF within tumors, but the absolute number of IFN-γ- and TNF-expressing CD8^+^ T cells was markedly increased upon combined T_reg_ cell ablation and IL-10R blockade, reflecting a combination of increased T cell infiltration and cytokine production (Fig. 7B and C). Similarly, the combination of T_reg_ cell depletion and IL-10R blockade resulted in an increase in the frequency and absolute number of IFN-γ- and TNF-expressing CD4^+^ T cells (Fig. 7D and E). These findings demonstrate that T_conv_ cells within tumors adopt IL-10 dependent suppressive activity upon therapeutic elimination of T_reg_ cells. This contributes to treatment failure of T_reg_ cell depleting immunotherapies and that combined targeting of T_reg_ cells and compensatory IL-10-dependent suppression invoked upon their depletion may enhance therapy.

## Discussion

T_conv_ cells and T_reg_ cells share components of their activation programs to meet similar metabolic, proliferative and migratory requirements as they transition from quiescent states to activated states (58–60). A substantial effort is now underway to develop therapies that specifically target molecules that distinguish T_reg_ cells within tumors from their T_conv_ cell counterparts. These efforts have been informed by comparative analyses of the molecular profiles of T_reg_ cells and T_conv_ cells within tumors under steady-state conditions (61, 62). We compared the transcriptional profiles of T_reg_ cells with T_conv_ cells not only under steady-state conditions, but upon immune activation provoked by experimental T_reg_ cell ablation. This analysis revealed that T_conv_ cells adopt a highly similar transcriptional profile to T_reg_ cells upon T_reg_ cell depletion. The extent of this reprograming goes beyond what would be expected as a result of the shared properties of T_reg_ cell and T_conv_ cell core lymphocyte activation programs and reveals that T_conv_ cells take on compensatory IL-10-dependent suppressive function when T_reg_ cells are eliminated. Acquisition of a T_reg_ cell-like transcriptional profile by T_conv_ cells upon T_reg_ cell ablation suggests that in practice there are very few molecules whose targeting will enable highly specific depletion of T_reg_ cells within tumors. Nevertheless, a small cluster of genes was identified in our analyses which has an expression profile limited to T_reg_ cells compared with T_conv_ cells under both steady-state conditions and upon T_reg_ cell depletion. While this cluster of genes may contain targets for specific depletion of T_reg_ cells within tumors, a question raised by this study is whether specific depletion of T_reg_ cells is indeed desirable, rather than the targeting of molecules shared by T_reg_ cells and cells with compensatory suppressive function induced upon T_reg_ cell depletion.

It is known that CCR8 marks highly suppressive T_reg_ cells found in both mouse and human tumors (40–44, 63). There is significant interest in the development of therapies which deplete CCR8^+^ T_reg_ cells within tumors. Given our observation that CCR8 also marks T_conv_ cells whose suppressive function is induced upon experimental T_reg_ cell ablation *in vivo*, it is reasonable to postulate that depletion of CCR8-expressing cells is a superior approach to T_reg_ cell depletion using other T_reg_ cell-expressed markers for induction of anti-tumor immunity, since CCR8-depleting therapies would target both T_reg_ cells and suppressive T_conv_ cells for destruction. It will be important to consider effects of CCR8-depleting therapies on both T_reg_ cells and CCR8^+^ T_conv_ cells within tumors, both in pre-clinical investigations (42, 63), and if robust intratumoral depletion of CCR8^+^ cells in the human clinical context is achieved. Alternatively, our data suggests that combining T_reg_ cell-targeted immunotherapies with blockade of IL-10 signaling will overcome compensatory suppression by T_conv_ cells.

It is interesting that the CCR8^+^ T_conv_ cell subset observed in human tumors did not phenotypically overlap with previously described Tr1 cells within tumors. Suppressive Tr1 cells have been characterized in several human tumors including head neck and squamous cell carcinoma (HNSCC) (64), colorectal cancer (52, 65), hepatocellular carcinoma (49), Hodgkin lymphoma (66), metastatic melanoma (67) and non-small-cell lung cancer (52), and their presence is often associated with tumor progression. The suppressive subset of CCR8^+^ T_conv_ cells we observe does not express EOMES or granzyme K, markers previously reported to be indicative of Tr1 cells. Recent studies have described a contribution of tumor-infiltrating follicular helper T (TFH) cells and follicular regulatory T (T_FR_) cells to anti-tumor immunity (68, 69). However, CXCR5 was not expressed by CCR8^+^ T_conv_ cells suggesting their distinction from T_FH_ cells. We did however observe high levels of GATA3 and *Il10* expression among CCR8^+^ T_conv_ cells, suggesting that they represent a Th2-like subset expressing high levels of markers associated with T cell activation, including CD25, expanded systemically and within tumors upon T_reg_ cell depletion. Prior work are consistent with these data, showing systemic expansion of Th2 cells expressing either GATA3 or Th2 cytokines upon experimental T_reg_ cell ablation (70, 71), and the intratumoral presence of CD4^+^ T_conv_ cells expressing CCR8 (40–44, 63). We found that the frequency of CCR8^+^ CD25^+^ FOXP3^−^ T_conv_ cells inversely correlates with the frequency of tumor-infiltrating CD8^+^ T cells, suggesting that they play an inhibitory role in human tumor immunity. The observation that CCR8^+^ T_conv_ cells expand and undergo activation to express IL-10 following depletion of T_reg_ cells provides an explanation of how bulk T_conv_ cells from tumors of T_reg_ cell-depleted animals acquire IL-10-dependent suppressive function despite lack of a change in the relative frequency of CCR8^+^ cells within the tumor CD4^+^ T_conv_ compartment. While our pre-clinical data from mouse models suggests that CCR8^+^ T_conv_ cells expand numerically within tumors upon experimental T_reg_ cell ablation, this is difficult to formally assess in humans due to the lack of specific markers that are differentially expressed in comparison with FOXP3^+^ T_reg_ cells, enabling their isolation *ex vivo*, and of clinically approved T_reg_ cell-depleting therapies in widespread use. However, a number of T_reg_ cell-targeted therapies are under development and it will be an important topic of future investigation to determine their effect upon CCR8^+^ T_conv_ cells in humans. It will also be important to better define how CCR8^+^ T_conv_ cells contribute to immune regulation in other contexts including infection and inflammation, as clones of Foxp3^-^ CD4^+^ T cells expressing CCR8, CD25 and IL-10 have previously been described in mice with experimental pulmonary granulomata (72), while CD4^+^ T_conv_ cells expressing CCR8 are observed upon experimental allergic lung inflammation in mice (73), and infiltrating human skin (74).

Our findings show that in the context of T_reg_ cell depleting immunotherapies, IL-10 production by T_conv_ cells represents a secondary layer of immune suppression responsible for driving immunotherapy resistance. It is important that this immunosuppressive function of IL-10 in limiting the efficacy of T_reg_ cell targeted therapies is appreciated, since in other contexts, IL-10 has been shown to possess immunostimulatory activity both in pre-clinical models and in clinical trials (75–77). Our findings suggest that IL-10 blocking antibodies may be used as a synergistic therapy with T_reg_ cell-targeted immunotherapies to improve patient outcomes.

Preclinical studies suggest that T_reg_ cell depletion can reinvigorate T_conv_ cell responses, however clinical trials of T_reg_ cell depleting therapies have thus far been met with limited clinical efficacy (35, 78). While our analysis of human tumor-infiltrating Foxp3^−^ T_conv_ cells revealed a fraction of CCR8^+^ T_conv_ cells under steady-state conditions, it will be valuable to examine whether such cells are expanded upon immunotherapy with either novel T_reg_ cell-depleting therapies, or anti-CTLA-4 therapy, the therapeutic efficacy of which is postulated to in part depend upon depletion or blockade of the suppressive function of T_reg_ cells (79, 80), and indeed in the context of non-T_reg_ cell-targeted immunotherapy approaches. The prognostic significance of such cells in determining the outcome of the immunotherapy responses will reveal insights into their broader contribution to immunotherapy resistance.

## Materials and Methods

### Study design

The objective of this study was to understand how T_reg_ cell depletion affects the function and immunoregulatory capacity of the T cell lineage in the context of tumor immunity. We used the well-established *Foxp3*^EGFP-DTR^ mouse to experimentally deplete T_reg_ cells in mice with syngeneic B16-F10 melanoma heterotopic tumors. We examined the consequences of T_reg_ cell depletion for tumor progression, as measured by blinded serial caliper measurements and tumor immunity, as assessed by scRNA-Seq and flow cytometry. We found that T_conv_ cells acquire T_reg_ cell-like suppressive functions upon depletion of T_reg_ cells. Using transcriptional profiling and *in vitro* suppression assays to better understand the nature of this suppressive activity, we found that this suppressive function was enriched among a Th2-like T_conv_ cell subset marked by expression of CCR8. Moreover, using antibody blockade and conditional *Il10* deletion experiments, we found that the suppressive activity induced upon T_reg_ cell depletion was dependent upon IL-10. The sample size for each experiment is specified in the figure legends. The number of independent experiments performed is stated in the figure legends. Age-matched male and female mice were randomly assigned to each group.

### Mice

*Foxp3*^EGFP-DTR^ mice were originally described by Kim et al. (36), *Foxp3*^IRES-EGFP^, *Ptprc*^a^ (CD45.1), and *Rag2*^−/−^ mice were obtained from the Jackson Laboratory. *Il10*^flox/flox^ *and Cd4*^Cre^ mice (81) were obtained from Jean Langhorne (Francis Crick Institute) and crossed with *Foxp3*^EGFP-DTR^ mice to generate *Il10*^flox/flox^ *Cd4*^Cre^ *Foxp3*^EGFP-DTR^ animals. Experiments were performed using 8-14 week old mice, with male and female mice equally distributed between experimental and control groups. Mice were housed at the University of Cambridge University Biomedical Services (UBS) Gurdon Institute Facility and Babraham Institute Biological Services Unit (BSU). Experiments were conducted in accordance with UK Home Office guidelines and were approved by University of Cambridge Animal Welfare and Ethics Review Board or by the Babraham Research Campus Animal Welfare and Ethics Review Board.

### Human primary tissues

Primary tumors and adjacent healthy tissue were acquired from 48 NSCLC patients. Patients gave consent to be included in the study which was approved by the institutional review board of Humanitas Research Hospital (protocols no. 2578). Patients did not receive chemotherapy, radiotherapy or palliative surgery before samples were obtained. Samples were processed using the gentleMACS Dissociator (Miltenyi Biotec) into single cell suspensions as previously described (82), resuspended in dimethylsulfoxide (DMSO) with 10% Fetal Bovine Serum (FBS) and stored in liquid nitrogen.

### High-dimensional flow cytometry analysis of human samples and computational processing of flow cytometric data

Samples were prepared for flow cytometry as previously described (82). Panels were developed according to an established protocol (83). Briefly, Flow Cytometry Standard (FCS) 3.0 files were analysed by standard gating in FlowJo version 9 to remove dead cells and cell aggregates, and identify CD4^+^ FOXP3^−^ T cells. 5,000 CD4^+^ FOXP3^−^ T cells per tumor sample (n = 48) were subsequently imported into FlowJo (version 10), biexponentially transformed, and exported in order to be analyzed by a custom-made publicly available pipeline of PhenoGraph (https://github.com/luglilab/Cytophenograph). A representative gating strategy is shown in Fig. S6. All samples were converted into comma separated value (CSV) files and concatenated in a single matrix by using the merge function of pandas package. The K value, indicating the number of nearest neighbors identified in the first iteration of the algorithm, was set at 500. Uniform Manifold Approximation and Projection (UMAP) was obtained by UMAP Python package.

### Tumor challenge and treatment

Mice were injected subcutaneously in the left flank with 1.25×10^5^ B16-F10 melanoma cells (ATCC). Tumors were measured at serial time points following implantation using digital calipers and tumor area was calculated as the length × width. Tumor measurements were completed by an independent investigator who was not aware of treatment groups or genotypes. For late DTx treatment experiments (starting day 10 post-tumor implantation), mice with tumors between 12 and 64 mm^2^ at day 10 were randomized to treatment groups to reduce experimental variability. Mice were injected intraperitoneally (i.p) starting at the indicated time point with 1 μg of diphtheria toxin (DTx) every other day for a total of four injections and/or 250 μg of anti-IL-10R (clone 1B1.3A; BioXcell) daily for a total of 10 days. DTx from *Corynebacterium diphtheriae* (Sigma-Aldrich) was obtained in lyophilized powder form and reconstituted in sterile double-distilled water according to the manufacturer’s instructions.

### Suppression assays

The suppressive capacity of tumor T_conv_ cells and T_reg_ cells was measured *in vitro* as previously described (84). Briefly, CD45.2^+^ TCRβ^+^ CD4^+^ GFP^+^ T_reg_ cells or CD45.2^+^ TCRβ^+^ CD4^+^ GFP^−^ T_conv_ cells were isolated from B16-F10 tumors of *Foxp3*^EGFP-DTR^ mice treated with PBS or DTx using florescence-activated cell sorting (FACS) 16 days post implantation and used as suppressor cells. A representative gating strategy is shown in shown in Fig. S2. Naïve CD4^+^ T_conv_ cells (CD25^−^ CD44^−^ CD62L^+^) were purified from the spleens of WT CD45.1 mice by FACS and stained with CellTrace Violet™ (CTV) according to the manufacturer’s protocol (Thermo Fisher Scientific) and used as responder T (T_resp_) cells. 2.5×10^4^ or 1.25×10^4^ (as indicated) suppressor CD4^+^ T_reg_ cells or T_conv_ cells were co-cultured with 1×10^5^ naïve responder T cells in the presence of anti-CD3 (BioLegend 1 μg/mL) and 5.0×10^4^ *Rag2*^−/−^ antigen presenting cells (APCs). T_resp_ cells cultured in the presence of anti-CD3 and APCs but without tumor T_reg_ cells or T_conv_ cells were used as a control. Cell division was evaluated by flow cytometry after 4 days of culture. For the screen, cells were cultured alone (gray) or with suppressors (purple) and with Aminoglutethimide (AG) at a final concentration of 125μM or with other indicated reagents at a final concentration of 10μg/mL.

### Flow cytometry of murine samples

Tumor samples were digested using collagenase and DNase for 30 minutes at 37 °C. Percoll was used to isolate lymphocytes from tumors. Tumors and spleens were mechanically dissociated over a 40 μm cell strainer. Red blood cells were lysed using ACK Lysing Buffer (Gibco). Cells were stained with the Fixable Viability Dye eFluor™ 780 (Thermo Fisher Scientific), Viakrome 808 (Beckman Coulter), or DAPI (Sigma) to discriminate between live and dead cells and then incubated with FC block (BioXcell, 2.4G2) the following surface antibodies for 30 minutes on ice: anti-TCRβ FITC (H57-597), anti-CD8 BV605 (53-6.7), anti-TIM-3 BV421 (RMT3-23), anti-CCR8 BV421 (SA214G2), anti-OX40 BV711 (OX-86), anti-TIGIT PE (4D4/mTIGIT), anti-ICOS BV750 (C398.4A), anti-LAG-3 BV785 (C9B7W), anti-CD3ε Spark Blue 550 (17A2) from Biolegend, anti-CD25 PE-Cyanine7 (PC61.5), anti-CD44 PerCP-Cyanine5.5 (IM7), anti-CD45.1 APC (A20), anti-CD4 PE-Cy7 (RM4-5) from eBioscience, and anti-GITR BUV805 (DTA-1), anti-CD4 BUV395 or BUV496 (GK1.5) from BD Biosciences. Cells were stimulated with phorbol 12-myristate 13-acetate (PMA) and ionomycin and blocked with brefeldin A (BFA) for 4 hours in RPMI 1640 complete medium. The intracellular antibodies anti-Foxp3 APC (FJK-16S), anti-IFN-γ PerCPCy5.5 (XMG1.2), anti-GATA3 PE-eFluor610 (TWAJ) were purchased from eBioscience, and anti-CTLA-4 BV605 (UC10-4B9), anti-TNF PE-Cy7 (MP6-XT22) were purchased from BioLegend and used with the eBioscience Foxp3/Transcription Factor Staining Buffer Set (Invitrogen, Thermo Fisher Scientific) according to the manufacturer’s protocol. For determination of CCR8 expression by Foxp3^−^ T_conv_ cells, cells from tumor-bearing *Foxp3*^EGFP-DTR^ animals were surface-stained and analyzed unfixed using flow cytometry, with EGFP expression used to discriminate T_reg_ cells and T_conv_ cells. Samples were analyzed using BD Fortessa, Beckman Coulter CytoFLEX, and Cytek® Aurora analyzers. After analysis, data were analyzed using FlowJo software (Tree Star, Inc.).

### scRNA Sequencing and analysis

Single cell suspensions of T cells were purified by total pan T cell enrichment (Invitrogen/Thermo Fisher Scientific), and live TCRβ^+^ cells were sorted from B16 tumors by FACS 16 days post implantation. RNA libraries were prepared for single cell RNA sequencing (scRNA-Seq) using the Chromium Single Cell 5’ Library & Gel Bead Kit v2 (10x Genomics) processed with Chromium (10x Genomics), and sequenced using the HiSeq 4000 System (Illumnia). Raw 10x sequencing data were processed as previously described and mapped to mm10. We confirmed that cells were sequenced to saturation. Data were merged with cell ranger aggr (cellranger-v5.0.0). Merged data were transferred to the R statistical environment for analysis primarily using the package Seurat (v3.2.2) in R v4.0.3. The analysis included only cells expressing between 200 and 2,500 genes, <5% mitochondrial-associated transcripts, and genes expressed in at least three cells. The data were then log-normalized and scaled per cell, and variable genes were detected using the FindVariableFeatures function in Seurat, as per default settings, using 2000 features and further processed as per the ScaleData function. Principal component analysis (PCA) was run on the variable genes, and the first six principal components (PCs) were selected for further analyses, based on the standard deviation of the PCs, as determined by an “elbow plot” in Seurat. Cells were clustered using the FindClusters function in Seurat with default settings, resolution = 0.5, and six PCs. UMAP was calculated using six PCs (RunUMAP function). For broadly defining the transcriptional features of each cluster, the FindAllMarkers function (only.pos = FALSE, min.pct = 0.1, thresh.use = 0.2, test.use = “MAST”) was used, and the associated heatmap was generated using the DoHeatmap function using up to the top 10 transcripts identified per cluster as defined by FindAllMarkers. The transcriptomic score of a particular cluster was calculated using the AddModuleScore function with default settings. Further visualizations of exported normalized data were generated using the Seurat RidgePlot functions and custom R scripts.

### RNA Sequencing and analysis

Single-cell suspensions were purified by FACS (as described above) 16 days post tumor implantation, and stored in 40 μl RNA*later*™ Stabilization Solution at -80 °C. RNA was extracted from samples using the RNeasy Plus Mini Kit (Qiagen) with optional QIAshredder step according to the manufacturer’s protocol. RNA-Sequencing (RNA-Seq) analyses were performed using ≥ 2 biological replicates. RNA-Seq was performed and analyzed as described previously (59). RNA Libraries were prepared using the Clontech SMARTer Ultra Low-input RNA kit (Takara) and sequenced on an Illumina HiSeq 2500 instrument using Illumina TruSeq v4.0 chemistry. The resulting FastQ files underwent quality control with FastQC, adaptor trimming with Cutadapt and alignment to the NCBIM37 *Mus musculus* genome annotation with hisat2 using ClusterFlow pipelines. Uniquely mapped reads were used to calculate gene expression and FPM values normalized to total library size with intergenic read normalization were calculated. Differential expression and statistical significance were calculated using the Wald test with adjustment for multiple testing using the Benjamini-Hochberg method using DESeq2 (85). Differentially expressed genes were further analyzed using R. PCA was performed using R *plotPCA* with count data transformed using variance stabilizing transformation (VST) from fitted dispersion-mean relationships generated using DESeq2 *vst*. Expression heatmaps were generated using FPM values normalized to row maxima using the R *pheatmap* package. Hierarchical clustering was performed using the Ward method. Dendrograms were cut at levels sufficient to allow 3-5 clusters to be discriminated.

### Statistical Analysis

Statistical analysis was performed using Graphpad Prism software. Two-tailed Student’s *t* tests or one-way ordinary ANOVAs were used as indicated to calculate statistical significance of the difference in sample means. *P* values of less than 0.05 were considered statistically significant. Statistical tests used are specified in the figure legends. In all figures, data represent the mean ± the standard error of the mean (SEM) or standard deviation (SD) as indicated. *P* values correlate with symbols as follows: ns = not significant, * *P* ≤ 0.05, ** *P* ≤ 0.01, *** *P* ≤ 0.001, **** *P* ≤ 0.0001.

## Supplementary Material

Data file S1

Data file S2

Data file S3

Data file S4

Data file S5

Data file S6

Supplementary Figures

## Data Availability

All bulk RNA-Seq and scRNA-Seq data will be made publicly available under NCBI Gene Expression Omnibus (GEO) accession number GSE236825. All other data needed to support the conclusions of the paper are present in the paper or the Supplementary Materials.

## One sentence summary

CCR8^+^ T_conv_ cells undergo expansion and activation upon T_reg_ cell depletion and limit anti-tumor immunity through production of IL-10.
