# Supplementary material for: Acquisition of suppressive function by conventional T cells limits anti-tumor immunity upon Treg depletion
Source: Sci Immunol. Author manuscript; Available in PMC 2024 Jan 5. (PMC7615475; doi:10.1126/sciimmunol.abo5558)
Supplement: Supplementary Figures [file EMS192862-supplement-Supplementary_Figures.pdf]

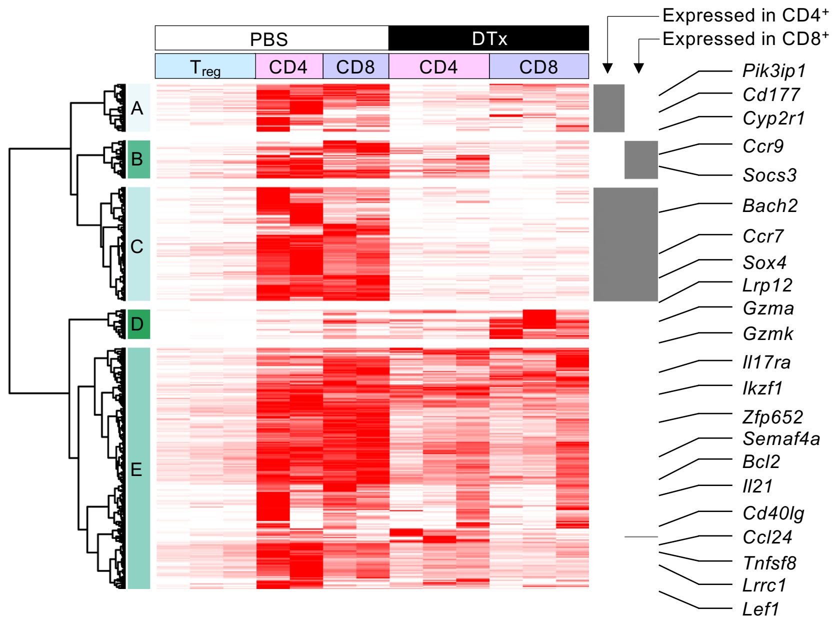

Figure S1. A majority of transcripts highly expressed within intratumoral T_{conv} cells compared with T_{reg} cells are downregulated within T_{conv} cells upon T_{reg} cell ablation. Heatmap showing the relative expression of transcripts downregulated ($q<0.05$; FC>4) in intratumoral T_{reg} cells compared with tumor $CD4^+$ T_{conv} cells in the indicated T cell subsets from tumors of B16-F10 tumor-bearing *Foxp3*$^{EGFP\text{-}DTR}$ animals administered either PBS or DTx. Colors indicate expression normalized to row maxima. *x*-axis hierarchical clustering identifies 5 clusters of genes with distinct expression patterns. Gray bars indicate expression that is less than a third the expression of transcripts in intratumoral T_{conv} cells under steady-state conditions.

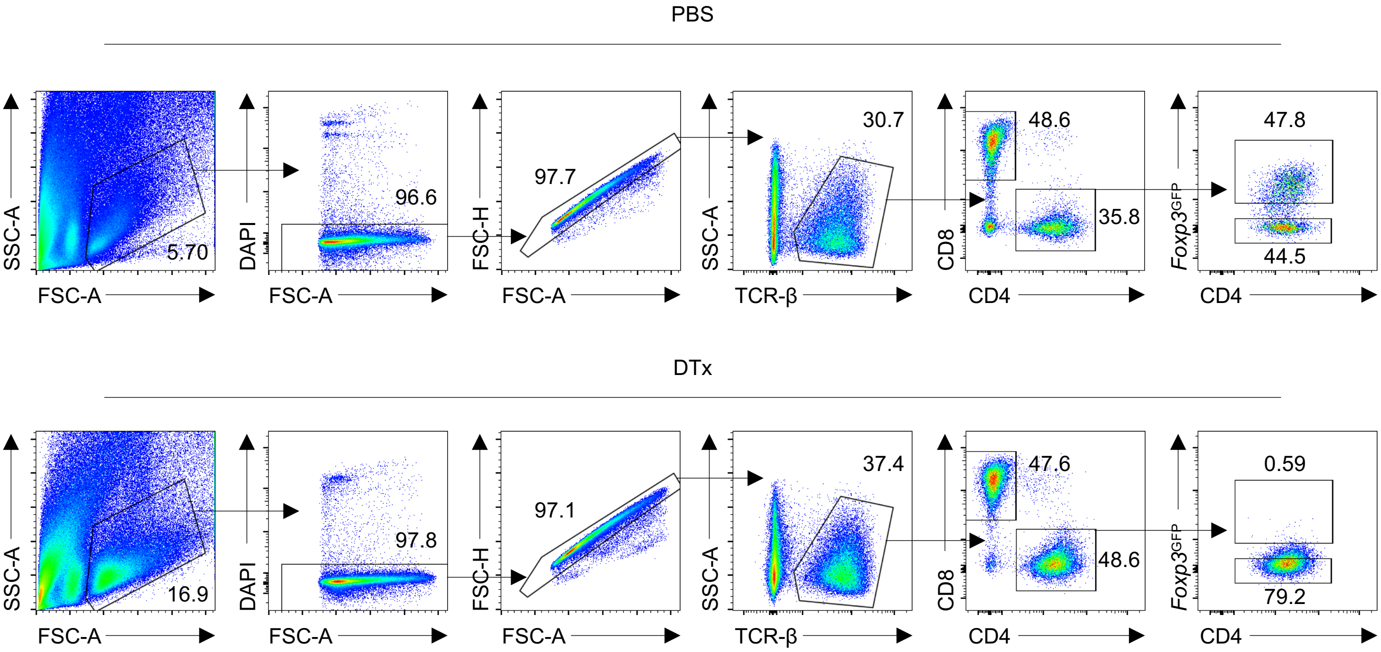

Figure S2. Isolation of $CD4^+$ $Foxp3^-$ T_{conv} cells from tumors of murine samples.

Representative gating strategy showing $CD4^+$ $Foxp3^-$ T cells. Numbers are percentages of cells within gate.

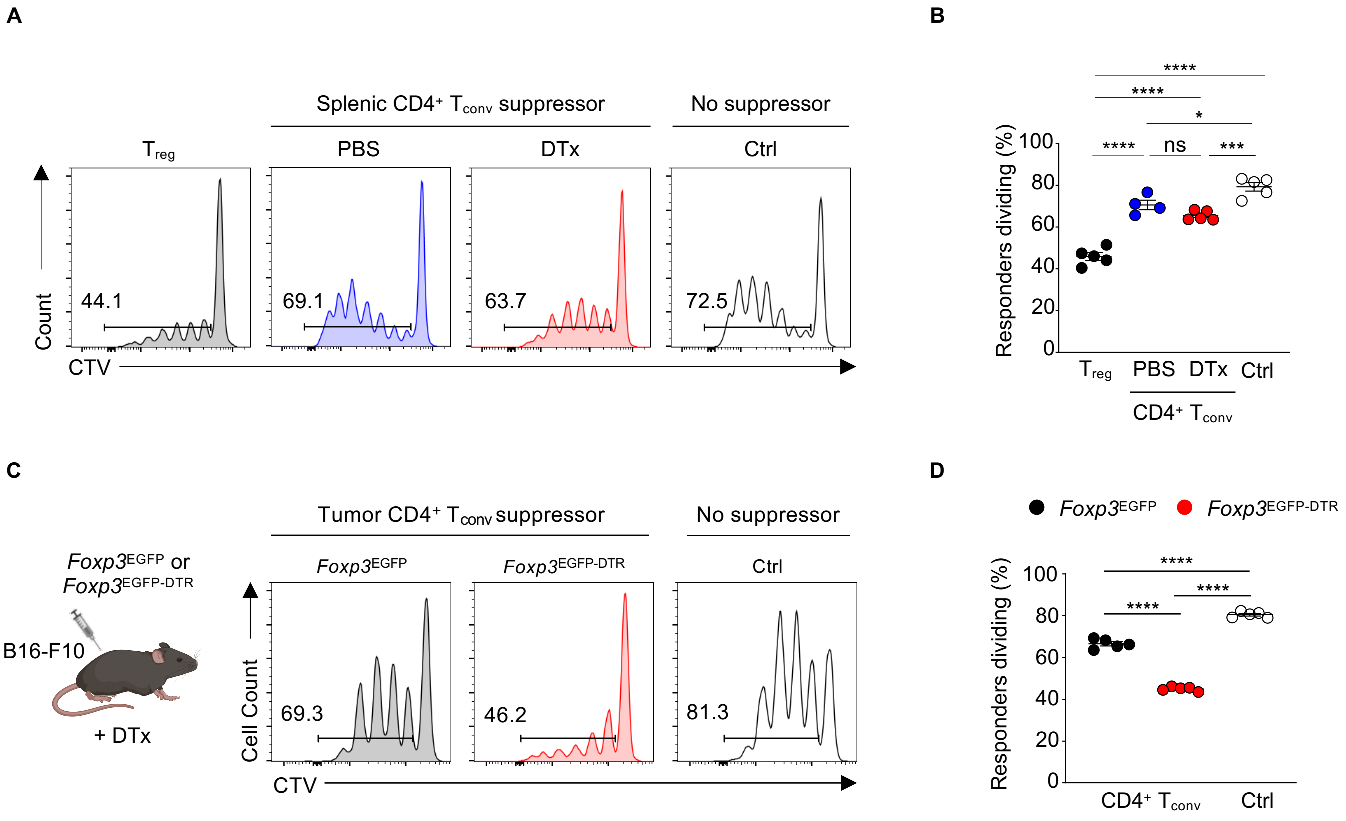

Figure S3. Increased suppressive function of $CD4^+$ T_{conv} cells from Treg cell-depleted mice. (**A**) B16-F10 cells were subcutaneously implanted into *Foxp3*$^{EGFP\text{-}DTR}$ mice and cells were isolated from spleens of PBS- and DTx-treated animals at day 16 post-implantation. $CD45.2^+$ $TCR\beta^+$ $CD4^+$ GFP^+ T_{reg} and $TCR\beta^+$ $CD4^+$ GFP^- T_{conv} cells were sorted by FACS from spleens and used as suppressor cells. Suppressor cells were co-cultured with responder T (T_{resp}) cells (naïve $CD45.1^+$ $CD4^+$ T_{conv} cells) at a ratio of 1:4, with 2.5×10^4 suppressor $CD4^+$ T_{reg} or T_{conv} cells co-cultured with 1×10^5 T_{resp} cells in the presence of 5.0×10^4 antigen-presenting cells (APC). $TCR\beta^+$ $CD4^+$ GFP^+ T_{reg} suppressor cells were sorted by FACS from mice treated with PBS only and used as controls. Representative histograms of dividing $CD45.1^+$ responder cells incubated with splenic T_{reg} or T_{conv} cells are shown. Naïve T_{conv} cells without splenic T_{reg} or T_{conv} cells were used as a control. (**B**) Replicate measurements of data shown in (**A**). Data are representative of > 4 independently repeated experiments, n < 4 per group. ****p <0.0001; ns, not significant; ordinary one-way ANOVA , Tukey's multiple comparisons. Bars and error show mean and s.e.m. (**C**) Experimental schema and representative histograms of dividing $CD45.1^+$ responder cells incubated with tumor T_{conv} cells isolated at day 16 after implantation of B16-F10 tumors in *Foxp3*$^{EGFP\text{-}DTR}$ and *Foxp3*^{EGFP} animals treated with DTx. Naïve T_{conv} cells without tumor T_{conv} cells were used as control. (**D**) Replicate measurements of data shown in (**C**). Data are representative of >2 independently repeated experiments, n < 4 per group. ****p <0.0001; ns, not significant; ordinary one-way ANOVA, Tukey's multiple comparisons. Bars and error show mean and s.e.m.

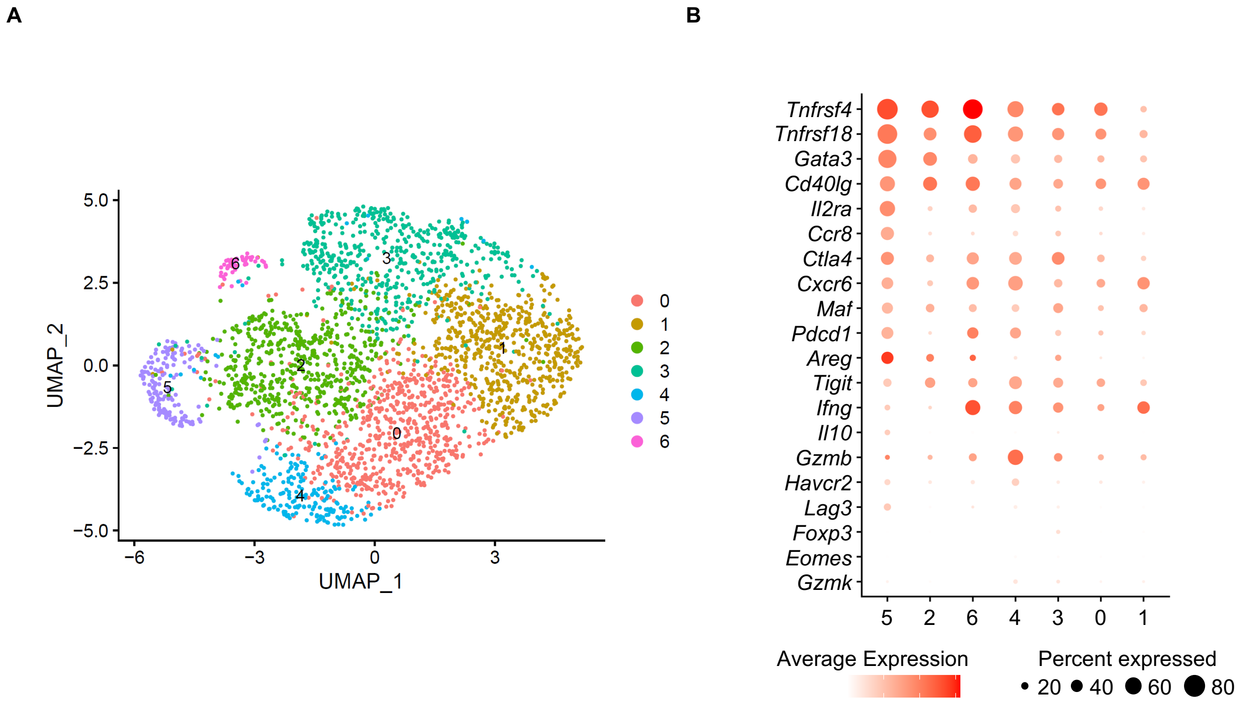

Figure S4. CD4^{+} T$_{conv}$ cells from T$_{reg}$ cell-depleted mice show an enrichment in expression of transcripts encoding for Th2-like and suppressive function. A. Uniform manifold approximation and projection (UMAP) of Cluster 0 sub-clusters. **B.** Bubble heatmap showing expression of indicated genes across subclusters of Cluster 0.

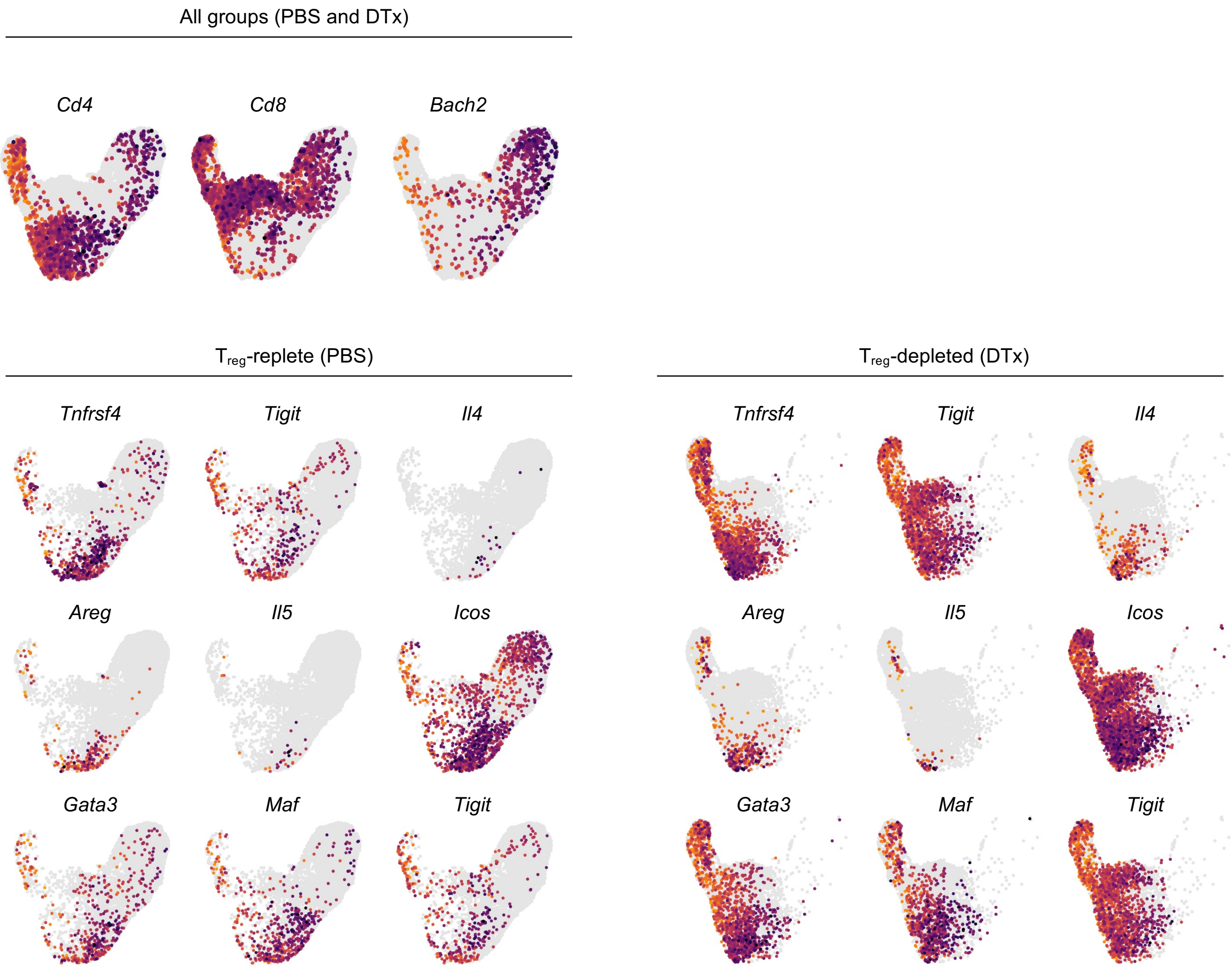

Figure S5. Markers largely expressed by intratumoral *Foxp3*$^{\mathrm{EGFP+}}$ T$_{\mathrm{reg}}$ cells become expressed by a subset of *Foxp3*$^{\mathrm{EGFP-}}$ CD4^{+} T$_{\mathrm{conv}}$ cells upon T$_{\mathrm{reg}}$ cell depletion. UMAP plots showing expression of indicated genes within T cells of tumors from tumor-bearing PBS- or DTx-treated *Foxp3*$^{\mathrm{EGFP\text{-}DTR}}$ animals.

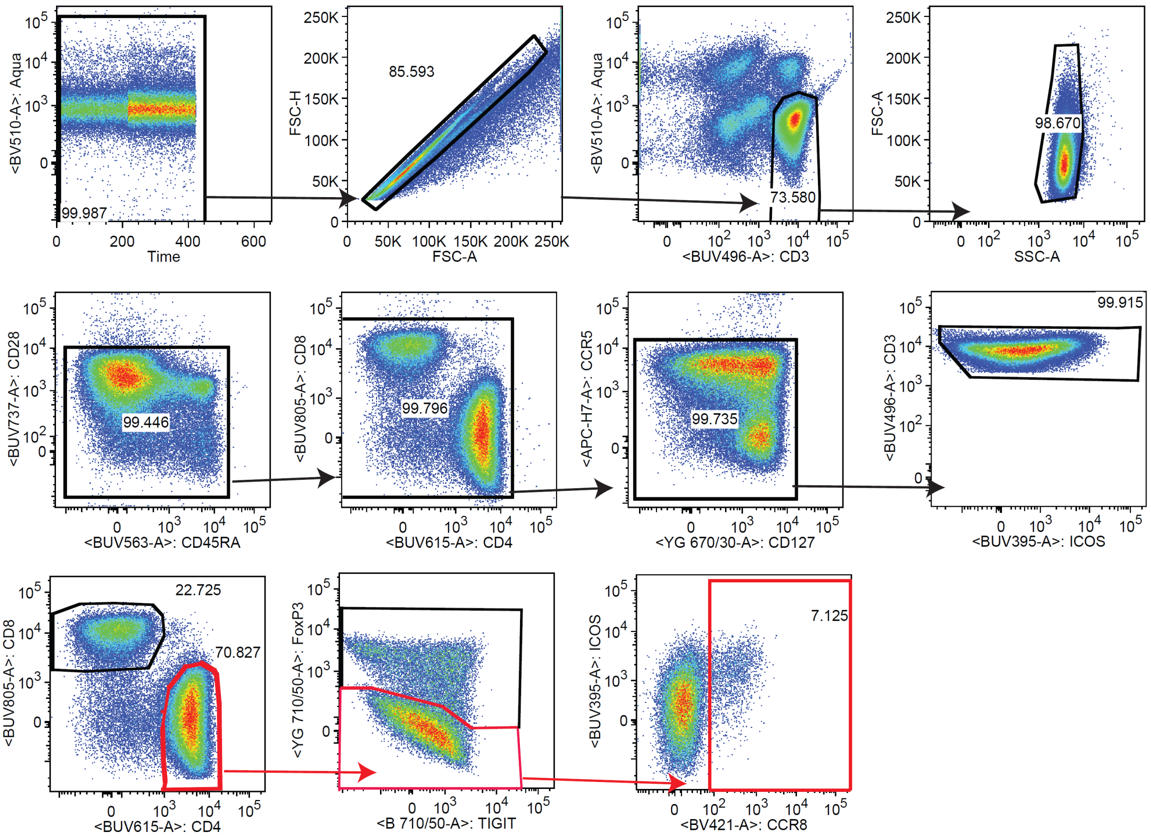

Figure S6. Isolation of CD4⁺ FOXP3⁻ T cells from tumors of NSCLC patients.

Representative gating strategy showing CD4⁺ FOXP3⁻ CCR8⁺ T cells from Fig. 5. Numbers are percentages of positive cells.

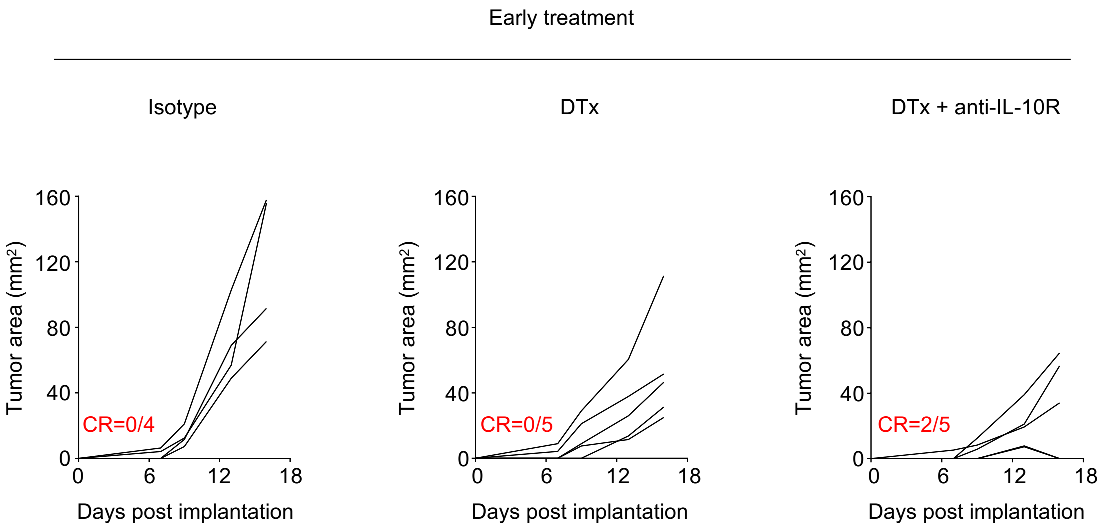

Figure S7. IL-10 blockade synergizes with early T_{reg} cell ablation to induce complete responses in a proportion of animals receiving combined therapy. Tumor area of heterotopic B16-F10 melanoma tumors at indicated time-points following implantation into *Foxp3*$^{EGFP\text{-}DTR}$ animals administered with indicated combinations of DTx and anti-IL-10R or isotype controls.
